# Air Sensor Network Analysis Tool: R-Shiny Application

**DOI:** 10.3390/atmos16111270

**Published:** 2025-11-08

**Authors:** Karoline K. Barkjohn, Todd Plessel, Jiacheng Yang, Gavendra Pandey, Yadong Xu, Stephen Krabbe, Catherine Seppanen, Renée Bichler, Huy Nguyen Quang Tran, Saravanan Arunachalam, Andrea L. Clements

**Affiliations:** 1 United States Environmental Protection Agency, Office of Research and Development, Research Triangle Park, NC 27711, USA; 2 General Dynamics Information Technology, Falls Church, VA 22042, USA; 3 Institute for the Environment, University of North Carolina, Chapel Hill, NC 27516, USA; 4 Applied Research Associates, Raleigh, NC 27615, USA; 5 United States Environmental Protection Agency, Region 7, Kansas City, KS 66101, USA

**Keywords:** air sensor, data analysis, data visualization, open-source software, air quality, air quality monitoring

## Abstract

Poor air quality can harm human health and the environment. Air quality data are needed to understand and reduce exposure to air pollution. Air sensor data can supplement national air monitoring data, allowing for a better understanding of localized air quality and trends. However, these sensors can have limitations, biases, and inaccuracies that must first be controlled to generate data of adequate quality, and analyzing sensor data often requires extensive data analysis. To address these issues, an R-Shiny application has been developed to assist air quality professionals in (1) understanding air sensor data quality through comparison with nearby ambient air reference monitors, (2) applying basic quality assurance and quality control, and (3) understanding local air quality conditions. This tool provides agencies with the ability to more quickly analyze and utilize air sensor data for a variety of purposes while increasing the reproducibility of analyses. While more in-depth custom analysis may still be needed for some sensor types (e.g., advanced correction methods), this tool provides an easy starting place for analysis. This paper highlights two case studies using the tool to explore PM_2.5_ sensor performance under the conditions of wildfire smoke impacts in the Midwestern United States and the performance of O_3_ sensors for a year.

## 1. Introduction

Poor air quality is associated with a variety of negative health effects [1]. Air quality data are needed to understand local conditions and reduce exposure to air pollution [2]. In the United States (U.S.), air quality is measured by ambient air monitors operated by state, local, and tribal air agencies [3]. Regulatory air monitoring includes federal reference method (FRM) and federal equivalent method (FEM) measurements [3]. Measurement technologies differ by pollutant, but FRM measurements are considered the gold standard for accuracy, with FEM measurements being equivalent in accuracy. These measurements provide information on the attainment of air quality standards in the U.S. along with a variety of other uses including real-time air quality communication to the public.

Air monitors do not capture all variation in air quality, especially during events like those related to wildfire smoke impacts, due to significant spatial and temporal variation [4,5]. Recent efforts aim to supplement the national monitoring network with localized air sensor data to investigate variations in air quality at neighborhood scales [6,7]. Air sensors typically cost one or two orders of magnitude less than conventional air monitors (e.g., USD 10-USD 10 k) and are designed to be compact, enabling additional measurements at more locations. However, air sensor data may be noisy [8], biased, or inaccurate [9]. Furthermore, sensor performance may vary over time [10,11], concentration range (e.g., nonlinear response), or the environment in which they operate (e.g., high relative humidity (RH), temperature) [12–14].

Limitations may be pollutant-, sensor technology- (e.g., optical, electrochemical, metal oxide), or manufacturer-specific. Quality assurance (QA) and quality control (QC) steps must be tailored for the specific sensor’s needs [15]. Particulate matter (PM) sensors are widely used, and fine particulate matter (PM_2.5_) is often highly correlated with reference measurements [9,16,17]. However, many of these sensors (e.g., Plantower, Sensirion) have limitations and only correctly measure particles from roughly 0.1 or 0.3 um up to 1 um with varying success depending on the sensor design [18–22]. While many of these sensors report particle counts and particles with diameters of 10 microns or less (PM_10_) in addition to PM_2.5_, they are often inaccurate [18,20,21,23]. Some PM sensors using optical particle counter (OPC) technology take PM_10_ and coarse particle (PM_10–2.5_) measurements that are strongly correlated with those of reference monitors [19,20,24]. Users must carefully consider specific sensor limitations.

The U.S. Environmental Protection Agency (EPA) has released air sensor performance targets and testing protocols for ozone (O_3_) [25], nitrogen dioxide (NO_2_), carbon monoxide (CO), sulfur dioxide (SO_2_) [26], PM_2.5_ [27], and PM_10_ [28]. These testing protocols recommend evaluating air sensors collocated with (i.e., running alongside) FEM and FRM measurements. Collocation studies have provided a lot of performance information about a variety of air sensor types over the past decade or more, allowing manufacturers, researchers, and other sensor users to better understand and improve sensor limitations [16,29–32]. In addition, correction equations are often required to generate accurate air pollutant concentration estimates [33]. Collocations have been used to develop local corrections for sensors [34–39] and to develop larger-scale (i.e., nationwide) air sensor correction when multiple collocations are considered in aggregate [40]. In recent years, sensor evaluations and corrections have increasingly been performed by opportunistically comparing sensors and monitors within a certain distance for local [41,42] or larger-scale (e.g., multi-state, regional) corrections [43–46] with some more complicated methods considering longer distances if sensor–monitor pairs are in areas with the same land use [47–49].

Recent work has explored the greatest needs related to air sensors. The U.S. EPA engaged in dialog with EPA Regions, and state, local, and tribal air monitoring agencies and found that many data users face challenges related to data management, analysis, and visualization [50]. The proposed solutions to these challenges included data analysis and visualization tools [51]. In a recent review, authors highlighted the need to bring together multiple sources of air quality information for comparison and the importance of data scientists partnering with data users when developing new tools [52]. Users without extensive coding experience face challenges in analyzing data from air sensors due to the complexities of accounting for sensor limitations, and many agencies are struggling with increased data volumes and more community questions all while dealing with steady or decreasing staff time.

Numerous tools exist for processing and analyzing air quality data, including R (v 4.5.1) [53–59], Python (v 3.13.7) [60–62], web-based tools [63,64], and Excel [65] tools in addition to commercial paid solutions. Many existing air data analysis tools require coding experience for operation in an open-source environment [54–57]; target specific manufacturers’ sensors [53,55]; or focus on evaluation based on a single collocation site [62,65]. An analysis tool is needed to more easily aggregate data from multiple air quality data sources, perform standard QC, identify nearby sensors spatially for comparison, and create comprehensive visualizations.

In response to these needs, the Air Sensor Network Analysis Tool (ASNAT) has been developed as an R-Shiny application designed to empower air quality professionals. This tool facilitates the evaluation of air sensor data quality through comparison with nearby measurements, supports the application of basic QA and QC, and enhances the understanding of local air quality conditions. By streamlining the analysis process, ASNAT enables agencies to quickly harness air sensor data for diverse applications, fostering reproducibility and reliability in air quality analysis. This tool was developed alongside the Air Sensor Data Unifier (ASDU) [66] which helps users to reformat, average, and apply basic quality control to air sensor data for later use in ASNAT and other tools.

This paper presents a detailed examination of ASNAT’s capabilities, illustrated through two case studies: (1) exploring sensor performance under the conditions of the Canadian wildfire smoke impacts in the Midwestern U.S. in June 2023 and (2) exploring the performance of O_3_ sensors at several sites within the U.S. from August 2019 to July 2020. Through these case studies, we demonstrate how ASNAT can enhance air quality monitoring and response strategies, ultimately potentially contributing to reduced exposure to air pollution, improved public health outcomes, and environmental protection.

## 2. Materials and Methods

### 2.1. Overview of ASNAT Functionality

ASNAT is an R-Shiny [67,68] application integrating data from multiple air quality networks (e.g., national monitoring network, sensor networks, meteorological stations) to provide a broader representation of local air quality. This tool was developed mostly in R with some code in C++ to enhance performance speed. It was designed to support air quality professionals with a base knowledge of air quality analysis to develop sensor data corrections, apply data flagging, understand the performance of air sensor networks, improve sensor data quality, and improve the comparability between networks so that users can better understand local air quality—including during extreme events like wildfires, dust storms, and fireworks.

Currently ASNAT has six tabs providing different ways to interact with the data. These include the map, flagging, tables, plots, corrections, and network summary tabs. After selecting data, users can decide to either move to the flagging tab or to the tables tab. In many cases, users may need to generate tables and plots before deciding what kind of flagging and removal of problematic data is needed.

The user manual, which can be downloaded along with the code, provides examples and walks through how to proceed with data analysis on each tab. Virtual user training has been provided to many users, and the training materials are available from the ASNAT team by request (i.e., email). Additional materials are under development.

### 2.2. Data for ASNAT

Users can load data from a variety of sources into ASNAT (version released 27 October 2025) including data from EPA’s Remote Sensing Information Gateway (RSIG) (https://www.epa.gov/hesc/web-access-rsig-data, accessed on 28 February 2025) [69], from PurpleAir (Draper, UT, USA) offline csv files, or from standard format files (as documented in the user manual), making it possible to use this tool for a wide variety of data types. Data can be loaded at hourly or 24 h averages, and NowCast averaging can be applied to visualizations and statistics within ASNAT (https://usepa.servicenowservices.com/airnow/en/how-is-the-nowcast-algorithm-used-to-report-current-air-quality?id=kb_article&sys_id=798ba26c1b1a5ed079ab0f67624bcb6d, accessed on 20 June 2025). The tool is designed, and was tested, to work with a variety of sensor and monitoring data with a focus on National Ambient Air Quality Standards (NAAQS) pollutants (i.e., PM_2.5_, PM_10_, O_3_, NO_2_, CO, and SO_2_).

Standard format files can be generated using ASDU [66] or through other means. ASDU allows the user to reformat text or csv data files from air sensors and air monitors, which currently come in a wide variety of formats for a variety of pollutants, into the standard ASNAT format by specifying column contents, time stamp formatting, sensor locations (i.e., latitude and longitude), and other formatting features. Users can save reformatting information to simplify loading data of the same type in the future [66]. During ASDU design and testing, 20 text-based file formats from 17 different manufactures were used to ensure ASNAT and ASDU could be used for a wide variety of sensor types [66]. If users can convert data into the standard ASNAT format using ASDU or other means, then they can analyze it in ASNAT, making ASNAT a flexible tool for analyzing data from various manufacturers, sensor models, and pollutants.

**Figure 1.**
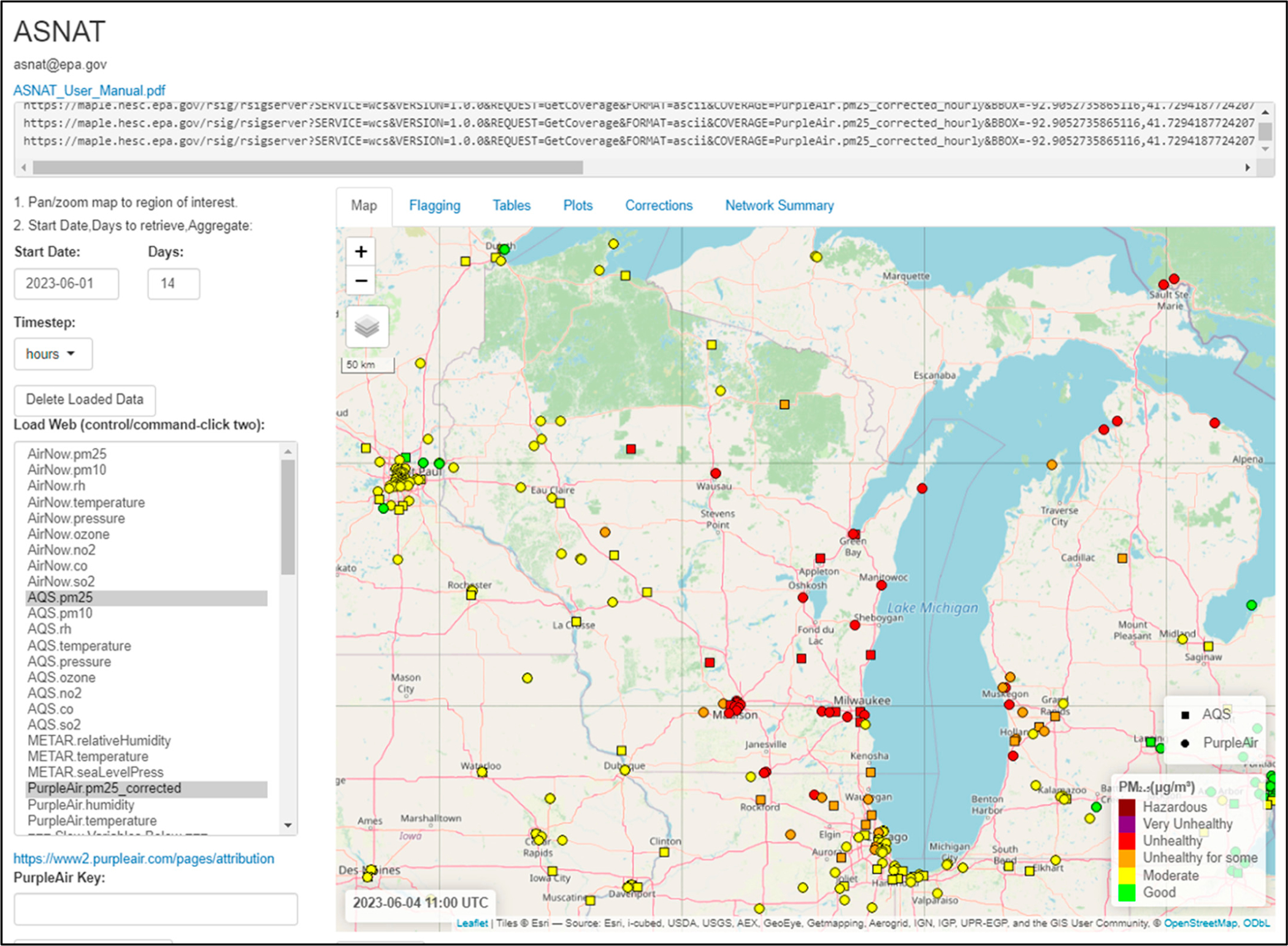
A screenshot of ASNAT, showing data selection on the left and loaded data displayed on the map on the right. The display includes AQS and corrected PurpleAir sensor data across the Midwestern U.S. and has multiple tabs to further quality-assure, summarize, and visualize the data.

Users can select data from RSIG (under “Load Web” in Figure 1), which allows users to efficiently call data based on a geographic bounding box. Users can retrieve AirNow (https://www.airnow.gov/, accessed on 20 October 2025), EPA’s Air Quality System (AQS) (https://www.epa.gov/aqs, accessed on 20 October 2025), Meteorological Aerodrome Report (METAR) (https://aviationweather.gov/data/metar/, accessed on 20 October 2025), and public crowdsourced PurpleAir data (https://www2.purpleair.com/, accessed on 20 October 2025). A full list of variables currently available in ASNAT through RSIG is included in the SI (Table S1). Users select the time period and pollutant or other variables of interest. After users load data, they can further refine their dataset of interest on the flagging tab (e.g., remove specific time periods, remove specific sensor or monitor IDs).

Data from AirNow and AQS available in ASNAT from RSIG include PM_2.5_, PM_10_, RH, temperature, pressure, O_3_, NO_2_, CO, and SO_2_. AirNow data are used to report the air quality index (AQI) to the public in near real time with data delivered at the end of every hour. In contrast, AQS data undergo more extensive quality control and are certified every quarter. This rigorous process ensures that AQS data can be used for regulatory purposes, such as determining the attainment of the NAAQS (https://www.airnow.gov/about-the-data/, accessed on 17 September 2025).

While the AQS and AirNow datasets are similar, there are some differences in availability and content. AQS data will typically become available a few months after collection due to the data certification process. When a user selects data from AirNow or AQS through the “load web” selection in ASNAT, RSIG requests the most recent data from AQS and AirNow, enabling access to the latest information. In some cases, more data may be included in the AirNow dataset as not all monitors also report to AQS. Depending on the application, AirNow or AQS data may be more appropriate, and users can easily run analysis with either and compare. In addition, users can specify which of the AirNow/AQS parameter codes for PM_2.5_ they would like, including 88101 (i.e., federal equivalent methods used for determining compliance with the PM_2.5_ National Ambient Air Quality Standards), 88500 (i.e., total atmospheric PM_2.5_), 88501 (i.e., PM_2.5_ raw data), or 88502 (i.e., acceptable PM_2.5_ AQI and Speciation Mass).

PurpleAir sensor owners can make the data public or keep it private. Public sensor data are taken from PurpleAir’s application programming interface (API) to EPA’s RSIG and are accessible in ASNAT. All PurpleAir variables are available in RSIG and ASNAT, although some are not accurate (e.g., PM_10_) [15,18,20,21,23].

Sensor owners include individuals; community groups; state, local, and tribal air agencies; schools; and others. For PurpleAir, data is sent from PurpleAir sensors connected to Wi-Fi to the PurpleAir API every 2 min. This data is then collected from PurpleAir’s API and saved in RSIG every 2 min and is then immediately available within ASNAT. PurpleAir hourly and daily aggregate files (i.e., one file per day per variable at 1 h and 24 h averages) are created a few minutes after midnight each day—so on a 24 h delay from real time. This means that requests for (pre-computed) pm25_corrected_hourly (as ASNAT provides) are only available for the previous day and before. This delay is due to the considerable processing time needed to create these (and other) archived files, so this is performed starting at midnight.

The PurpleAir dataset includes the corrected PurpleAir PM_2.5_ data (PurpleAir.pm25_corrected) corrected in a similar way as PurpleAir data on the AirNow Fire and Smoke Map (fire.airnow.gov, accessed on 28 January 2025) including completeness criteria (75% for daily or hourly averages), exclusion when A and B channel measurements disagree (i.e., data are excluded if both |A − B| > 5 µg/m^3^ and (|A − B| × 2)/(A + B) > 70%), and the application of the U.S.-wide correction, accounting for nonlinearity at high smoke concentrations (Equation (1)). Here, PA_cfatm_ is the atm correction of the PurpleAir data, and RH is the relative humidity. This method may be updated in the future if improvements are made.


(1) $${\mathrm{PA}}_{\mathrm{cfatm}}<30:\;{\mathrm{PM}}_{2.5}=[0.524\;\times {\mathrm{PA}}_{\mathrm{cfatm}}]-[0.0862\;\times \;\mathrm{RH}]\;+\;5.75\;30\;\leq {\mathrm{PA}}_{\mathrm{cfatm}}<50:\;{\mathrm{PM}}_{2.5}=[0.786\;\times ({\mathrm{PA}}_{\mathrm{cfatm}}/20\;-\;3/2)\;+\;0.524\;\times \;(1\;-({\mathrm{PA}}_{\mathrm{cfatm}}/20\;-\;3/2))]\;\times {\mathrm{PA}}_{\mathrm{cfatm}}-\;[0.0862\;\times \;\mathrm{RH}]\;+\;5.75\;50\;\leq {\mathrm{PA}}_{\mathrm{cfatm}}<210:\;{\mathrm{PM}}_{2.5}=\;[0.786\;\times {\mathrm{PA}}_{\mathrm{cfatm}}]-\;[0.0862\;\times \;\mathrm{RH}]\;+\;5.75\;210\leq {\mathrm{PA}}_{\mathrm{cfatm}}<260:\;{\mathrm{PM}}_{2.5}=\;[0.69\;\times ({\mathrm{PA}}_{\mathrm{cfatm}}/50\;-\;21/5)\;+\;0.786\;\times \;(1\;-\;({\mathrm{PA}}_{\mathrm{cfatm}}/50\;-\;21/5))]\;\times {\mathrm{PA}}_{\mathrm{cfatm}}-\;[0.0862\;\times \;\mathrm{RH}\;\times \;(1\;-\;({\mathrm{PA}}_{\mathrm{cfatm}}/50\;-\;21/5))]\;+\;[2.966\;\times ({\mathrm{PA}}_{\mathrm{cfatm}}/50\;-\;21/5)]\;+\;[5.75\;\times \;(1\;-({\mathrm{PA}}_{\mathrm{cfatm}}/50\;-\;21/5))]\;+\;[8.84\;\times \;(10^{-4})\;\times \;{{\mathrm{PA}}_{\mathrm{cfatm}}}^{2}\times ({\mathrm{PA}}_{\mathrm{cfatm}}/50\;-\;21/5)]\;260\leq {\mathrm{PA}}_{\mathrm{cfatm}}:\;{\mathrm{PM}}_{2.5}=\;2.966\;+\;[0.69\;\times {\mathrm{PA}}_{\mathrm{cfatm}}]+[8.84\;\times 10^{-4}\times {{\mathrm{PA}}_{\mathrm{cfatm}}}^{2}]\;$$

Users must supply a valid PurpleAir API read key (available directly from PurpleAir) to access this data. Users can also load data from standard format text files, which can be generated using the ASDU tool (https://www.epa.gov/air-sensor-toolbox/air-sensor-data-tools, accessed on 28 February 2025), or from offline PurpleAir files from the SD card. These alternative methods are especially important for groups interested in analyzing private data.

METAR data available include temperature, RH, and sea level pressure. For METAR data, the files (hourly) are downloaded from a remote server to maple (internal EPA computer) via a cronjob every 15 min. No user interaction is involved. When a user requests METAR data such as hourly data for 7 days, it is aggregated and streamed on-the-fly based on reading the archived hourly METAR files.

Data are displayed in coordinated universal time (UTC) by default, but users can adjust the UTC offset on the first tab. Data loaded through RSIG are already time-aligned. Users can provide the time zone offset for data processed through ASDU into ASNAT to time-align the datasets. Users cannot adjust the time alignment between different data sources in ASNAT at this time.

### 2.3. Map Tab

Once users select and load data, monitoring sites appear on the map at the top of the map tab (Figure 1). Each data type is assigned a symbol, and users select a colormap that is then described in the legend. Once users have loaded hourly or daily averaged data, users can choose how to view the data (e.g., loaded hourly or daily averages, nowcast averages, mean value over all timesteps) and use the timestep slider below the map to animate the information, if applicable.

### 2.4. Tables Tab

Users can load one to three variables at a time for comparison. If the user has selected only one variable (i.e., X variable), the tables tab summarizes the data by site ID including the count (i.e., number of hours or number of days available), percent of missing data, mean, minimum, quartiles, and maximum.

Typically, selecting two variables involves comparing sensor data with more uncertainty and unknown performance to nearby reference monitor data (e.g., AirNow, AQS) to better understand sensor performance and develop corrections. Usually, the variable with more uncertainty would be the Y variable. If a user selects two variables, they will also specify the maximum distance threshold for comparison. Distances are calculated using Euclidean distance between latitude, longitude pairs. If there are no pairs within this distance, the user will see the error “There are no neighboring points within [x] meters” when they try to generate tables on the tables tab. Users must carefully consider the appropriate distance depending on the pollutant, local geography, meteorology, local sources, and other factors. If the user has selected two variables within a certain distance, it provides the same summary table for each variable. In addition, it provides a table of the neighboring points at each time stamp, which can be especially helpful if the user would like to save the data for further analysis outside of ASNAT, and a table summarizing each pair, including IDs, locations, distance between the pair, and the coefficient of determination (R^2^). Lastly it provides a summary of the paired data by AQI category. This includes the number of points in each AQI category (i.e., count), the normalized mean bias error (NMBE), root mean squared error (RMSE), and normalized root mean squared error (NRMSE) in each AQI category. This can help the user to understand how the bias and error between the two datasets change over the full range of the datasets.

Selecting three variables will allow the user to explore how a third variable (e.g., RH) influences the relationship between two other variables (e.g., sensor data and monitor data). If the user generates these tables and then uses the save data button, these summary tables will be saved along with the raw data.

### 2.5. Plots Tab

The plots tab includes additional visualizations to understand network performance and local air quality. ASNAT describes the relationship between the X and Y datasets (e.g., sensor–monitor relationship) through a series of performance metrics calculated as outlined in the EPA’s performance targets with different targets for different pollutants [25–28]. Each point in the boxplot represents a single sensor–monitor pair with at least two data points (e.g., two hourly averages, two daily averages). The target values on the plots are set based on the EPA’s performance targets and are not user-modifiable at this time.

Users can also explore the performance of the sensors by considering how frequently the sensors and monitors report the same AQI category. This type of analysis is important to understand whether sensors can be used to advise various health protective actions suggested in different AQI categories. There is no standard guidance to interpret differences, so users will need to use their best judgment depending on the application and averaging interval. NowCast and 24 h averages are more frequently used for AQI display, and it is typically more challenging for sensors and monitors to agree at shorter averaging intervals (e.g., hourly). This analysis can be performed at 1 h or 24 h or using NowCast averages.

### 2.6. Corrections Tab

Using the corrections tab, users can develop sensor corrections to adjust for bias between the sensor and the monitor (or whatever two comparison variables the user selects). Sometimes, a simple linear correction allows two datasets to become more comparable. However, much past work with air sensors has shown that RH or temperature can influence sensor measurements [12–14], and many gas sensors may have cross-sensitivities to other gases [13], so a third variable may be needed to account for some of these influences or interferences. In ASNAT, currently available corrections include single-variable, multivariable additive, or multivariable interactive corrections in either linear, quadratic, or cubic form. Users can generate corrections for individual sensors and then can export the corrections. The corrections can be applied and are shown on corrected scatterplots and a summary table on the corrections tab. Users can generate the different types of corrections and compare the provided statistics (i.e., R^2^, RMSE, NMBE) along with a visual assessment of the scatterplot to understand what, if any, correction works best for their dataset. Currently, users cannot apply corrections in ASNAT to data on other tabs and cannot apply user-generated corrections developed outside of the tool. ASNAT applies the following completeness criteria before generating corrections:

- Spatial validity: Sensor–monitor pairs must be neighbors within the user-specified maximum neighbor distance.
- Data quality: Only unflagged data (variable Y and variable X with flag status = “0”) are used in correction development.
- Data availability: All primary variables (X, Y) must have non-missing values (e.g., any paired X/Y row with a missing value for X or Y is removed); for multivariable models, the third variable (Z) is also required with at least 50% data completeness across matched rows (e.g., if three variables are selected, the Z variable is only included in the correction if it has 50% completeness with the non-missing matched X/Y data).
- Minimum sample requirements: ASNAT requires sufficient data points for correction. For basic model fitting and statistical validation (R^2^ calculation), a minimum of two records are required for single-variable models and >15 records for multivariable models. For coefficient generation, the minimum requirements vary by correction complexity: linear corrections require ≥20 records, quadratic corrections require ≥30 records, and cubic corrections require ≥40 records. These are the minimums for correction generation, but they do not ensure robust correction development. The user must examine the plots, examine correction performance, and consider whether the conditions during this period are representative of the conditions they plan to apply the correction over.

### 2.7. Network Summary Tab

The network summary tab allows users to understand local air quality using all sensors/monitors in the view area on the map selection or within a user-specified geoJSON (e.g., uploaded state or county boundary). A map can be generated showing the mean, maximum, or the difference between the mean and the concentration at each site (i.e., to highlight spatial variation). A bar plot is also created of the mean, maximum, or difference values. If PM_2.5_ or PM_10_ daily data are selected, a bar plot of daily AQI by category will also be made. The user can also generate a time series plot or two types of calendar plots. The network summary tab also allows users to compare two datasets with each other. These plots will show daily patterns by the hour of the day and day of the week and averages by the month of the year and a scatterplot of the two datasets compared. Data on this tab are different because the tab will compare all data within the selected geographic area instead of just pairs within the user-specified distance and will not remove flagged data. The user can then download all current plots and data frames.

### 2.8. Flagging Tab

The flagging tab provides a variety of options to flag and/or remove anomalous data based on several categories such as the following: the exceedance of a threshold, agreement with the nearest neighbor, repeated values, outlier detections using several methods (e.g., Hampel filter [70,71]), a user-specified period (e.g., a day with a known sensor issue), and more (Table S2). Although this is the second tab after the map tab, in many cases, users will need to return and flag data after identifying problems in the tables, plots, and potentially other tabs. In some cases, the data may not be anomalous, but users may want to remove it to answer specific questions. For example, users may want to remove specific sensors because the sensors were not operated by a data analyst (e.g., public sensors versus those run by a local agency) or the data are incomplete (i.e., large data gaps). PurpleAir data has a count column indicating how many values were used to generate the average; this can be used to flag data for incompleteness if users want to exclude averages below a certain completeness (e.g., 90% completeness = count > 27 for hourly data generated from raw 2 min PurpleAir data). More than one flag can be applied, and users can remove or reset all current flags. No automated recommendations are made, and users will need to use their best judgment to flag and remove data since in some cases, outliers may be localized events. This is one of the reasons this tool is targeted at users with some air quality experience.

Flags impact some plots and tables and can be exported. Performance plots on the plots tab (i.e., overall scatterplot, AQI agreement plot, boxplots of performance targets) are shown with all data, and only unflagged data and flagged points are indicated in lighter shades on the time series. When data are downloaded using the “Save Data” button, data will be saved and will have a “flagged(−)” column including all the current flags associated with each measurement. Flag numbers are separated by semi-colons. A value of 0 means unflagged, values of 80 and above are used for built-in conditions, and those 1–79 are reserved for user-defined Boolean conditions corresponding to the lines in the saved flags.txt file in the specified output directory. Users can press the “save flag conditions” button to export the flag conditions they entered as Boolean expressions for their records, but for other flagging, users may need to document the flags they used for reproducibility.

### 2.9. Case Study: 2023 Midwestern Smoke

We present a 2-week case study to demonstrate the features and functionality of this tool. During June 2023, the Midwestern U.S. was impacted by smoke from Canadian wildfires [72]. During uncommon events like these, state and local agencies may be interested in better understanding how sensors perform and whether any improved QA or QC is needed to provide accurate data to the public. Further, the synthesis of air quality data from multiple observational networks supports current and future agency response (e.g., publicizing current air quality conditions and suggested response, documenting evidence of exceptional events). For this example, PM_2.5_ data were loaded from RSIG, including AQS PM_2.5_ data and the PurpleAir corrected dataset from the bounding box shown in Figure 1 including all of Wisconsin (WI); most of Michigan (MI); parts of Minnesota (MN), Iowa (IA), Illinois (IL), Indiana (IN), and Ohio (OH); and a small piece of Ontario, Canada.

We considered distances between sensor–monitor pairs between 50 and 4000 m, in line with the comparison distances used in past work [42–46]. For each distance, we evaluated the number of AQS sites with sensors within various distances, the number of nearby pairs, and the overall R^2^ of all points and between each pair. We also considered the R^2^ overall and each pair after excluding the top quantile of the data (PurpleAir PM_2.5_ > 18 µg/m^3^) since R^2^ can be driven by one or more outlier points. Ultimately, we selected a distance of 250 m between sensor–monitor pairs for a more in-depth case study analysis.

Hourly PM_2.5_ data were used throughout. We considered the hourly performance of sensor–monitor pairs using the EPA’s performance targets for PM_2.5_ sensors [27]. In addition, we compared the AQI category provided by the corrected PurpleAir sensors to nearby air monitors.

### 2.10. Case Study: O_3_ Sensor Performance

We also present an evaluation of Aeroqual (Auckland, New Zealand) AQY v1 O_3_ sensors. The Aeroqual AQY is a multi-pollutant sensor measuring temperature, RH, dew point, NO_2_, O_3_, and PM_2.5_. The AQY uses the Aeroqual gas-sensitive semiconductor sensor to measure O_3_. The results presented here reflect out-of-the-box performance. We did not use Aeroqual’s calibration feature where users can conduct an initial colocation and input a slope and offset into the online dashboard, which could have further improved the sensor’s performance.

This sensor was evaluated as part of our long-term performance project [73]. During this project, a variety of sensors were collocated alongside air monitors at sites across the U.S. Sensors were within 20 m of the monitors, as specified in the EPA’s performance targets [25], with many sensors being within a few meters. Data were downloaded weekly by site operators and were reformatted and hourly averaged using ASDU and then loaded using the “Load data from a standard-format file” feature in ASNAT. AirNow O_3_ data was then loaded from RSIG using the “Load Web” feature. AirNow data, rather than AQS data, was used because the North Carolina (NC) collocation site is not a regulatory site and only reports to AirNow. This case study focuses on performance at a subset of sites (5 of 7 sites) included in the full project including NC, Arizona (AZ), Colorado (CO), Delaware (DE), and Oklahoma (OK) (Table 1, Figure S1). These sites represent a variety of environmental conditions potentially contributing to differences in sensor performance. Three sensors operated at the NC site with single sensors at the other sites. This case study focuses on a 1-year subset of the total evaluation period (i.e., excludes pre- and post-collocation periods in NC and extended collocation that occurred at some sites before Aug 2019 and after July 2020). Additional details on this study can be found in a previous publication on the project [73] and the Supplemental Information included with that publication.

We considered the hourly performance of sensor–monitor pairs using the EPA’s performance targets for O_3_ sensors [25]. Past work has shown that NO_2_ is not an interferent for AQY sensors [29] but that temperature and RH may influence measurements [74] and that measurements may drift over time [48]. For this reason, the temperature and RH data measured by the sensors were used to try to better correct the sensor data. In addition, a custom computed variable, days since deployment, was used to understand sensor drift. This was determined by calculating the number of days since 1 August 2019. Sensors had initial collocation periods and started sensing at slightly different times across the sites, but this is a good estimate of how drift may have impacted performance over the 1-year period considered during this analysis. This case study demonstrates the functionality of ASNAT for a sensor dataset that is not part of RSIG.

Some custom R code was written to support this analysis.

1. Custom code was used to sort files by headers as some files had minor differences that would not be accepted by ASDU.
2. Files were processed through ASDU in batches by time zone (i.e., eastern, central, mountain), and then custom code was used to concatenate and sort by time stamp to meet the requirements for ASNAT import.
3. AQY data was loaded from local files, and then AirNow data was loaded from RSIG with pairs within 1000 m used to match sensor data with the collocated monitoring sites. This was a huge dataset of AirNow sites across a large portion of the U.S. Custom code was used to remove all sites that were not matched with AQY sensor collocations to improve the speed of loading the data for further analysis. This step would not be required if all analysis was completed in a single ASNAT session or if users did not mind waiting for all data to be reloaded from AirNow.
4. Custom code was used to add a column of days since deployment started (i.e., 1 August 2019).

## 3. Results

### 3.1. Comparison Distances Considered for 2023 Midwestern Smoke

For the largest paired dataset (distance = 4000 m), the distribution of the corrected PurpleAir dataset shows an average = 16 µg/m^3^, median of 13 µg/m^3^, first quartile of 9 µg/m^3^, third quartile of 18 µg/m^3^, and maximum of 204 µg/m^3^. R^2^ values can be influenced by high-concentration points; therefore, we examined performance both including all data and excluding the highest quartile of data (>18 µg/m^3^) (Table 2).

When comparing neighbors within 50 m, only five AQS sites have PurpleAir sensors within 50 m, and there are nine neighbor pairs all in IA. Including all data, there is a strong R^2^ overall (R^2^ = 0.88) and for each pair (R^2^ = 0.80 − 0.99). When excluding the highest quartile of data, the data is still moderately correlated (R^2^ = 0.63), and each pair is moderately to strongly correlated (R^2^ = 0.48 − 0.95). Expanding the distance to 250 m identifies additional AQS sites (8 total) with additional neighbors (16 total). Correlations are similar, except over the full range, the R^2^ by pair ranges from moderate to strong (R^2^ = 0.62 − 0.99) instead of strong correlation across all pairs seen at 50 m. All pairs within 250 m are still located in IA.

Expanding to 500 m reveals additional cities in IL and MN with 11 AQS sites and 20 neighboring pairs total. The overall R^2^ values for all data are similar, but the R^2^ by sensor–monitor pair for the full range of concentrations ranges from weak to strong (0.27–0.99), and for the lower-concentration data, it ranges from no correlation to strong (0.03–0.95). At increased distances (i.e., 1000, 2000, 4000), additional pairs are identified, but correlations remain similar but with weak correlations seen for all low-concentration data for pairs within 4000 m (R^2^ = 0.03). We selected a distance of 250 m moving forward as further distances seem to have some sensors that are not well correlated to the monitor, suggesting localized sources or other issues leading to differences in sensors and monitors being further apart.

### 3.2. Nearby Sensor–Monitor Pairs for 2023 Midwestern Smoke

**Figure 2.**
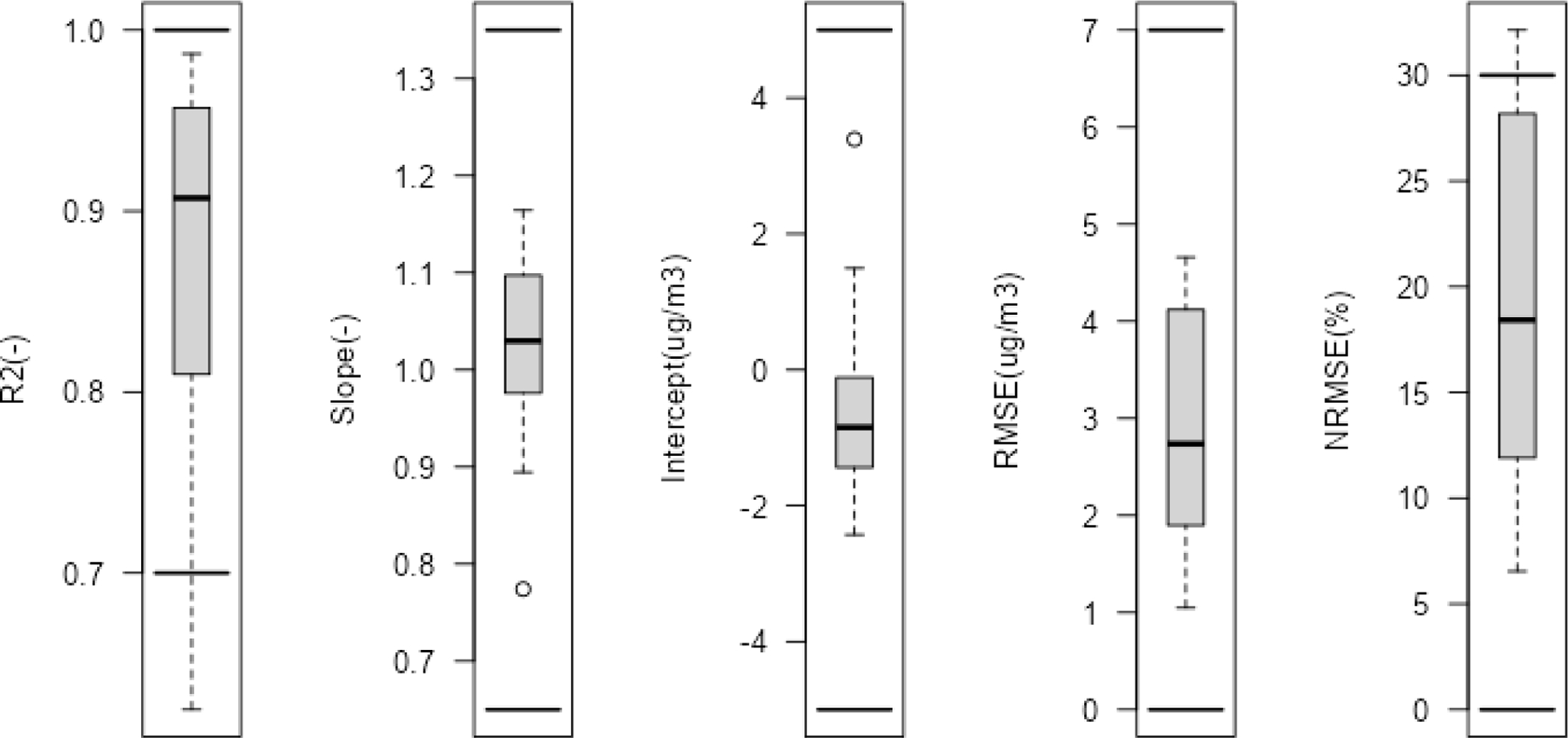
Performance of hourly corrected PurpleAir PM_2.5_ data as compared to nearby monitors (data from AQS) for 16 pairs within 250 m at hourly averages. Black lines indicate target range for each metric (coefficient of determination (R^2^), slope, intercept, root mean squared error (RMSE), and normalized root mean squared error (NRMSE)).

In this example, sensor–monitor pairs within 250 m were selected, resulting in 16 neighboring pairs. Most sensor–monitor pairs are strongly correlated, meeting the EPA’s performance target for the coefficient of determination (R^2^) with only a few pairs falling below the 0.7 target (Figure 2). All sensor–monitor pairs meet the targets for slope (1 ± 0.35), intercept (−5 ≤ b ≤ 5 mg/m^3^), and root mean squared error (RMSE ≤ 7 mg/m^3^). Some sensors have a normalized root mean squared error outside of the target (>30%); however, the EPA’s performance targets [27] only require meeting either the RMSE or the NRMSE target. Overall, sensors perform well during this time.

**Figure 3.**
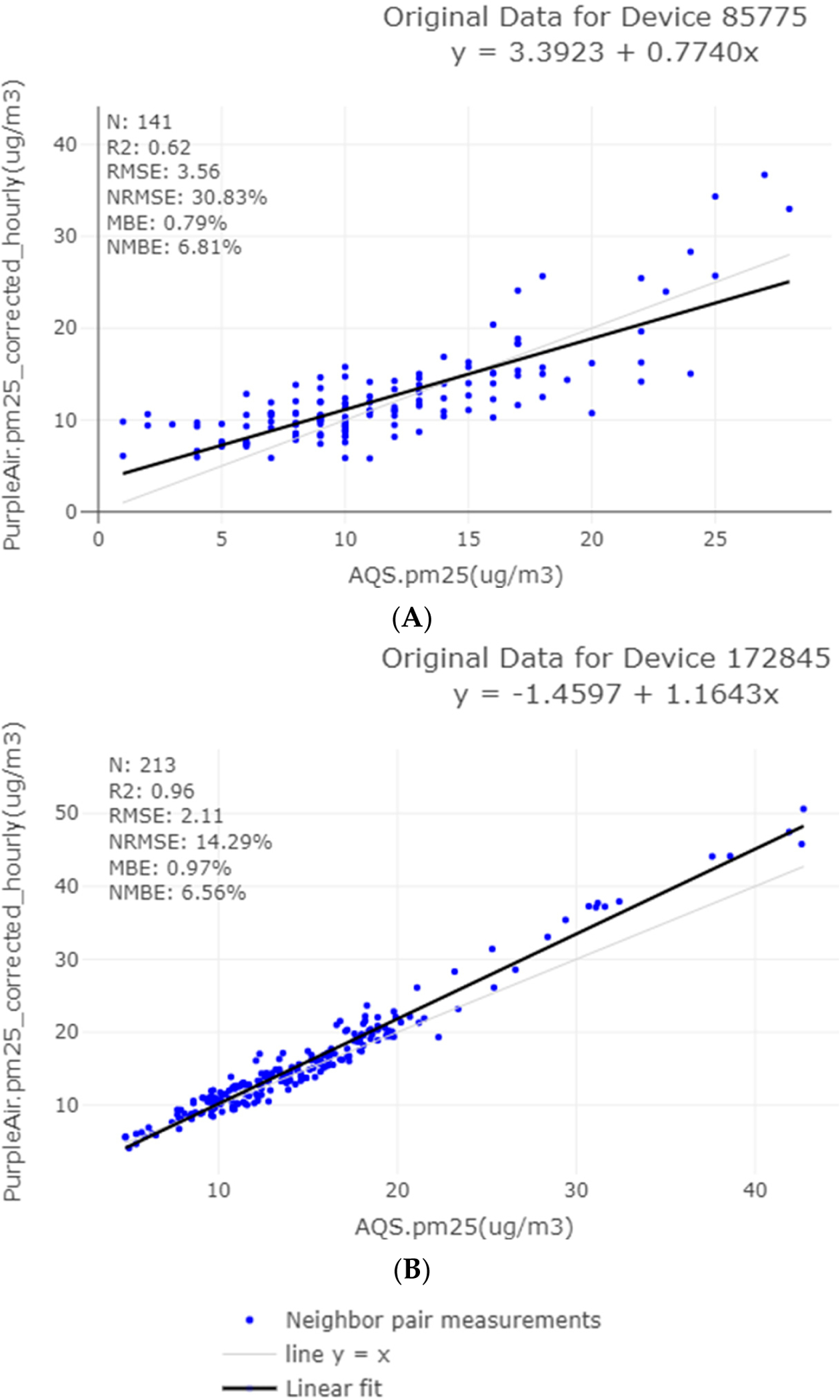
Corrections generated for hourly PM_2.5_ data from two example sensor–monitor pairs as visualized by ASNAT. The top plot (panel (**A**)) shows a sensor with a weak correlation. The bottom plot (panel (**B**)) shows a sensor with strong agreement.

Using the corrections tab, we dig into the performance of individual sensors. Figure 3A shows a sensor with a weak correlation (R^2^ = 0.62 < 0.7). There are a variety of reasons why this sensor may have weaker performance than the typical sensor during this period (Figure 2). For instance, the user may have improperly reported the latitude and longitude of the sensor, meaning that the sensor may be more than 250 m from the AQS monitor, differences in the federal equivalent method (FEM) may lead to differing performance, this sensor may have hardware or software differences leading to different performance, or there may be other issues. In addition, R^2^ is dependent on concentration range, so R^2^ may be lower in part since the range of concentrations experienced is smaller than other sensor–monitor pairs. While a correction is provided for this sensor (y = 4.26 + 0.73x), we typically would not apply it since the R^2^ is weak. This plot shows the “original data” for the device, but plots can also be generated showing the data after correction. ASNAT does not treat pairs with low correlation differently, but the user can remove these pairs in the flagging tab if they desire.

Figure 3B shows that a different sensor has strong agreement and low normalized mean bias error (NMBE) when compared to the nearby reference monitor. Depending on the project objectives, this performance may be adequate. If higher accuracy is needed, the individual sensor correction generated (y = −1.4597 + 1.1643x) could be applied.

**Figure 4.**
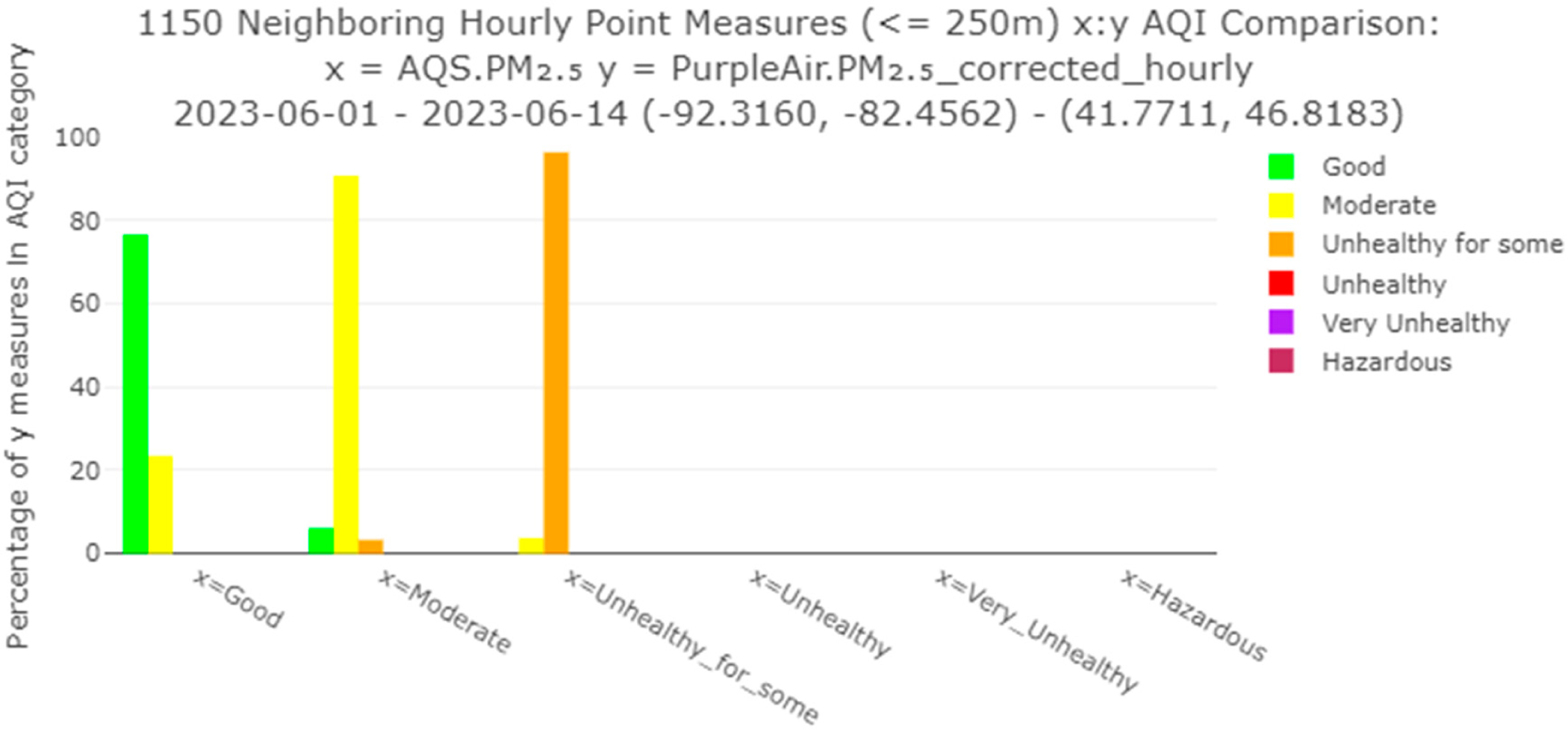
The percentage of hourly PurpleAir PM_2.5_ measurements in each AQI category compared to the monitor (AQS)-reported AQI category as visualized by ASNAT. Corrected PurpleAir typically reports the same AQI category as nearby monitors over our midwestern area (range of longitudes: −92.3160, −82.4562; range of longitudes: 41.7711, 46.8183).

Overall, the sensors in this example show strong AQI category agreement with sensors typically reporting the same AQI categories as monitors. Specifically, sensor and monitor hourly average AQI categories match at least 76% of the time across categories (Figure 4). When the AQS monitors report a good AQI, sensors within 250 m report a good AQI almost 80% of the time and moderate AQI just over 20% of the time. When the AQS monitors report a moderate AQI, sensors within 250 m also report a moderate AQI roughly 90% of the time but occasionally indicate a good or unhealthy AQI for sensitive groups (shortened to unhealthy for some in the ASNAT application) instead. Lastly, when the AQS monitors report an unhealthy AQI for sensitive groups, the sensors almost always also report an unhealthy AQI for sensitive groups but occasionally (<10% of the time) show a moderate AQI instead. These results indicate that corrected PurpleAir data can be reliably used to communicate AQI-associated health-protective actions to the public during a smoke event such as this example.

### 3.3. O_3_ Sensor Performance

**Figure 5.**
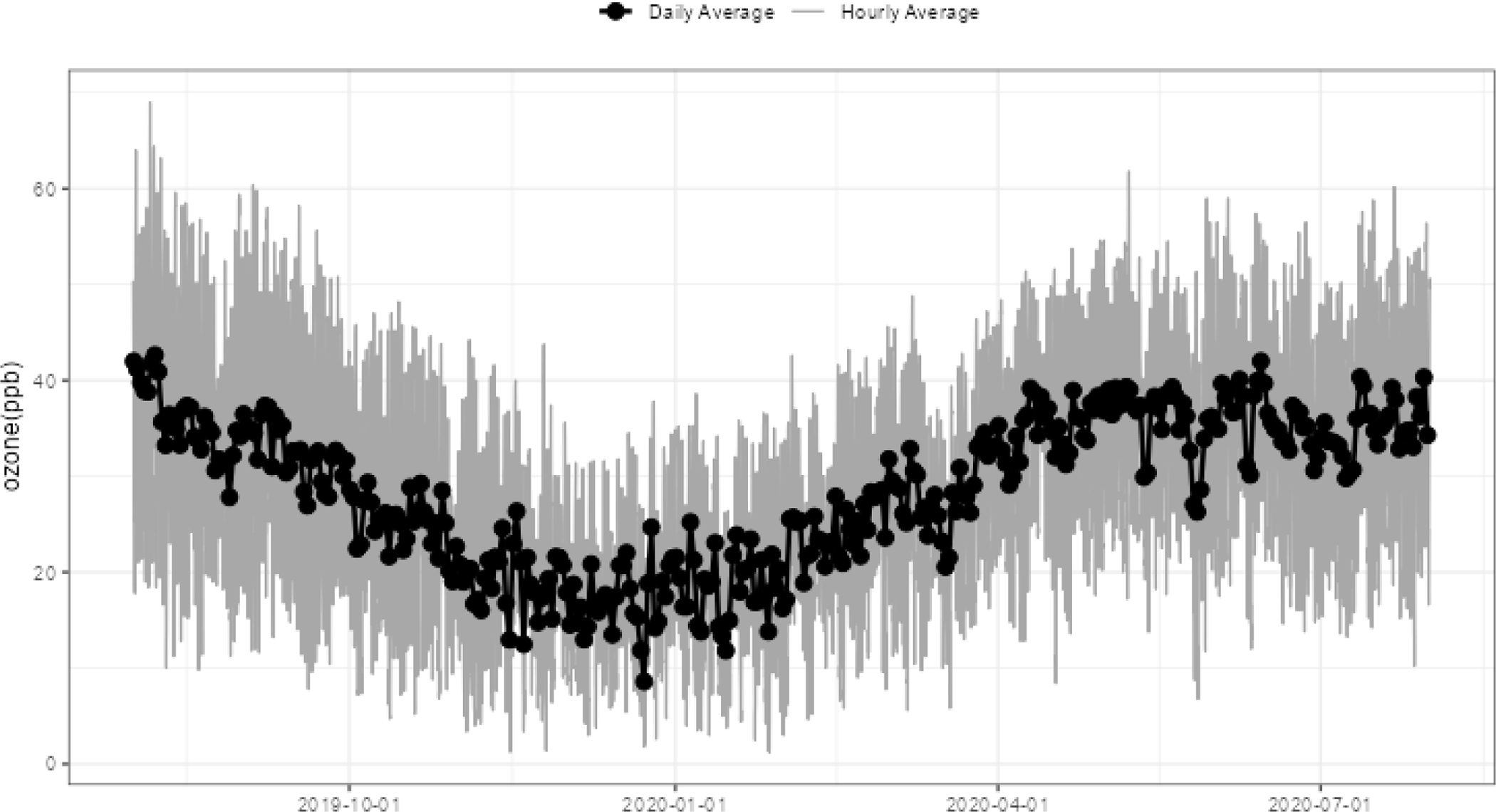
Ozone concentrations over the study time period as measured by the AirNow monitors as visualized in ASNAT.

**Figure 6.**
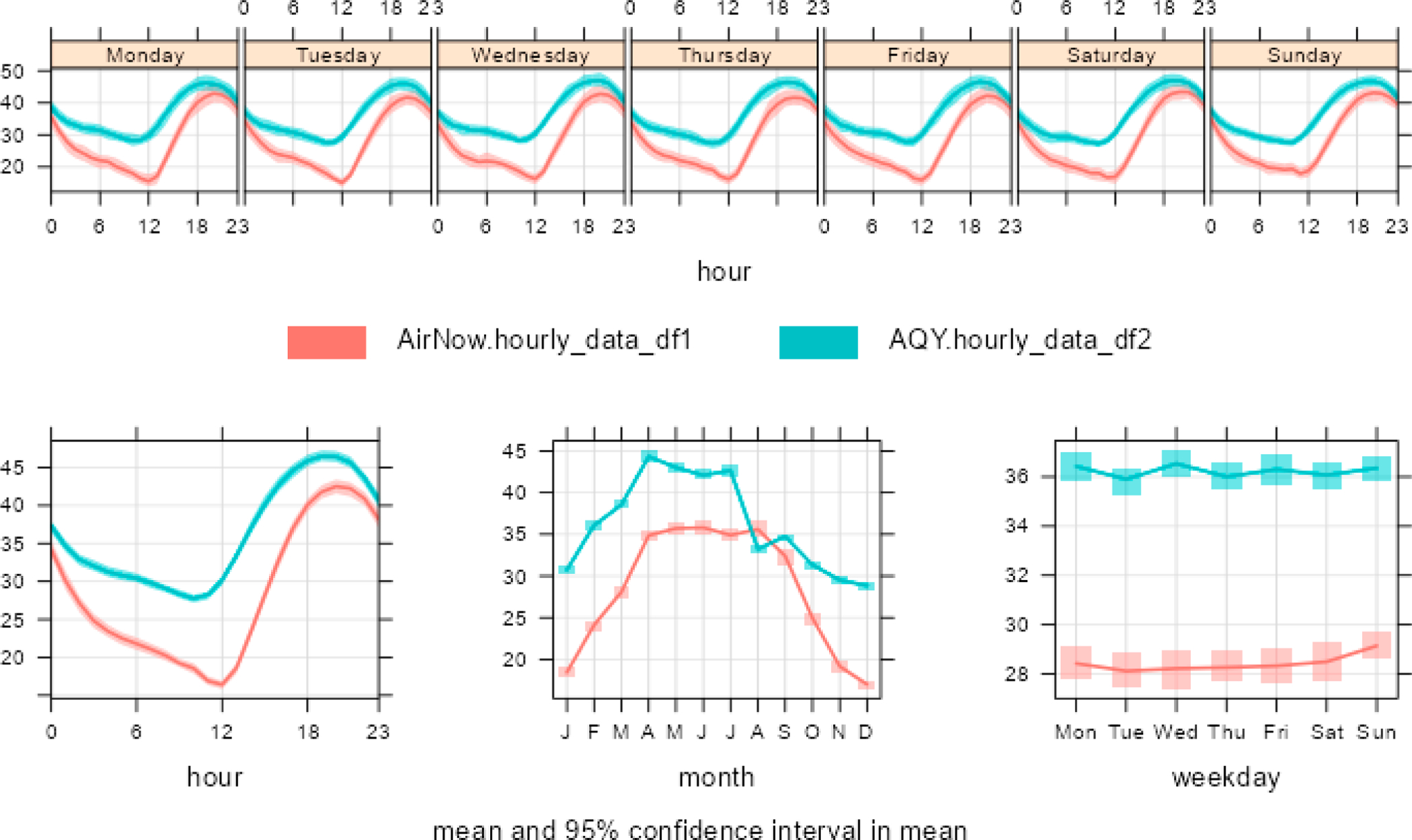
A visualization from ASNAT showing ozone concentrations over the study period as measured by the AirNow monitors (red) and the AQY sensors (teal). Months are abbreviated by the first letter of the month (e.g., J January, F February).

Ozone concentrations varied over the study year with higher concentrations in the summer and lower concentrations over the winter (Figure 5). Similar concentrations are seen across the sites (Table 1, Figure S2) with slightly higher average concentrations in OK (mean = 33 ppb; other sites = 27–28 ppb). AirNow monitors and AQY sensors show similar trends by hour of day and month of year with uncorrected AQY sensors typically overestimating O_3_ concentrations (Figure 6). Daily average concentrations do not vary largely over the course of a week, and the bias between sensors and monitors is consistent (Figure 6).

**Figure 7.**
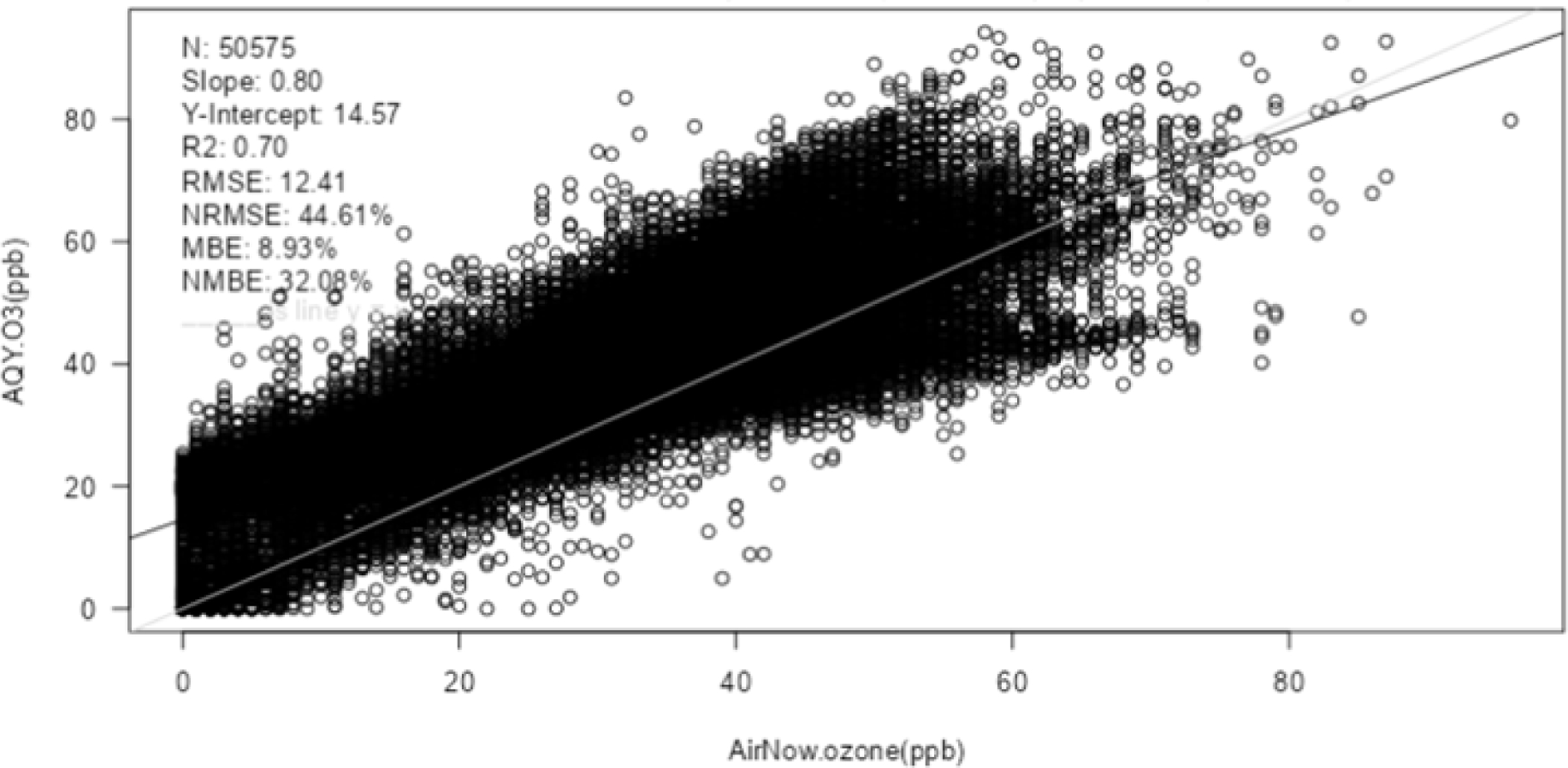
Hourly O_3_ sensor data compared to AirNow monitor data at 5 sites in AZ, CO, DE, NC, and OK from August 2019 to July 2020. The black line is the linear regression, and the light grey line is the one to one line.

**Figure 8.**
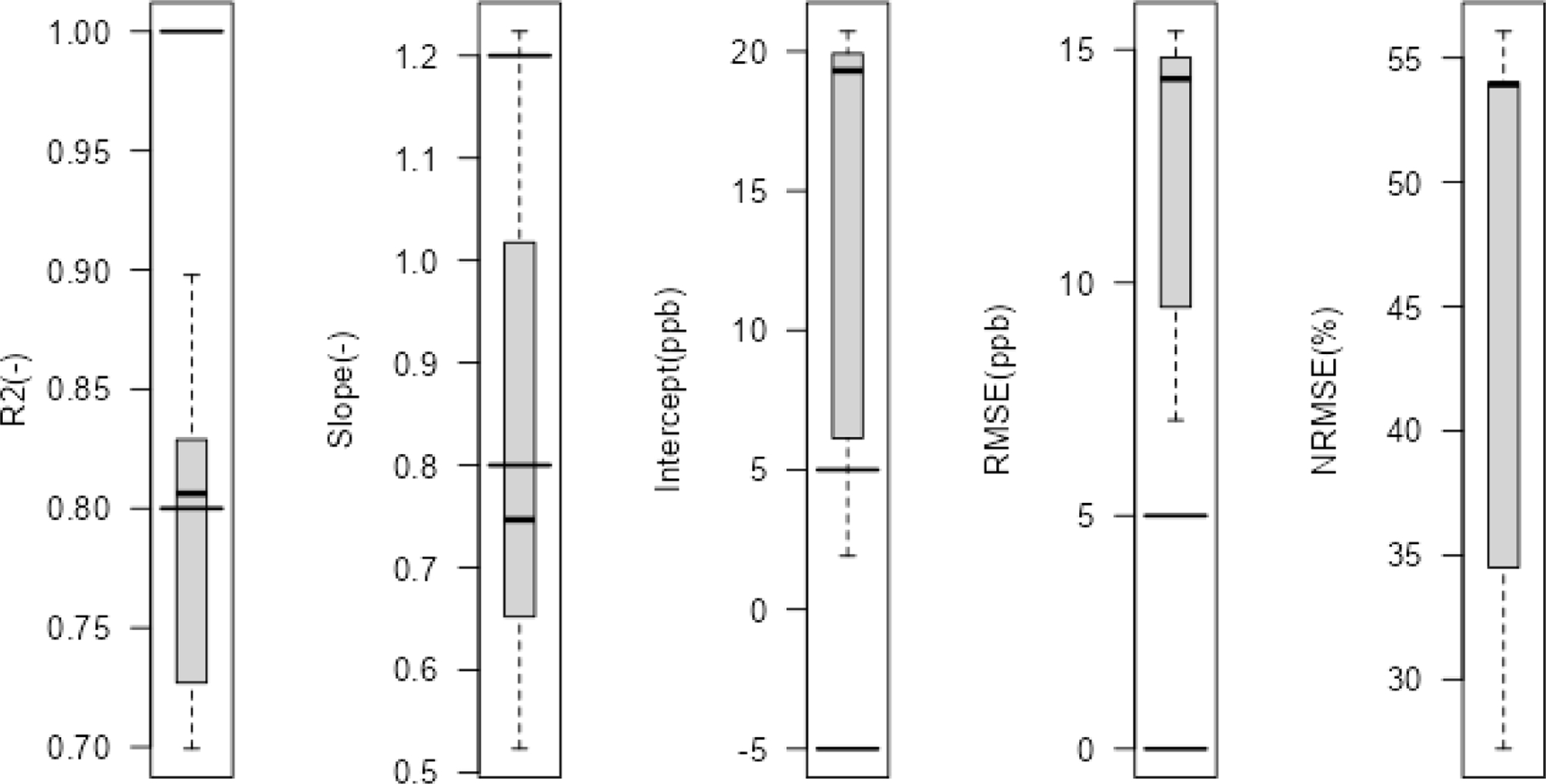
Hourly performance of AQY O_3_ sensors compared to AirNow monitors at 5 sites in AZ, CO, DE, NC, and OK from August 2019 to July 2020. The horizontal lines indicate the performance target ranges for O_3_ for each metric except NRMSE where there is no target.

**Figure 9.**
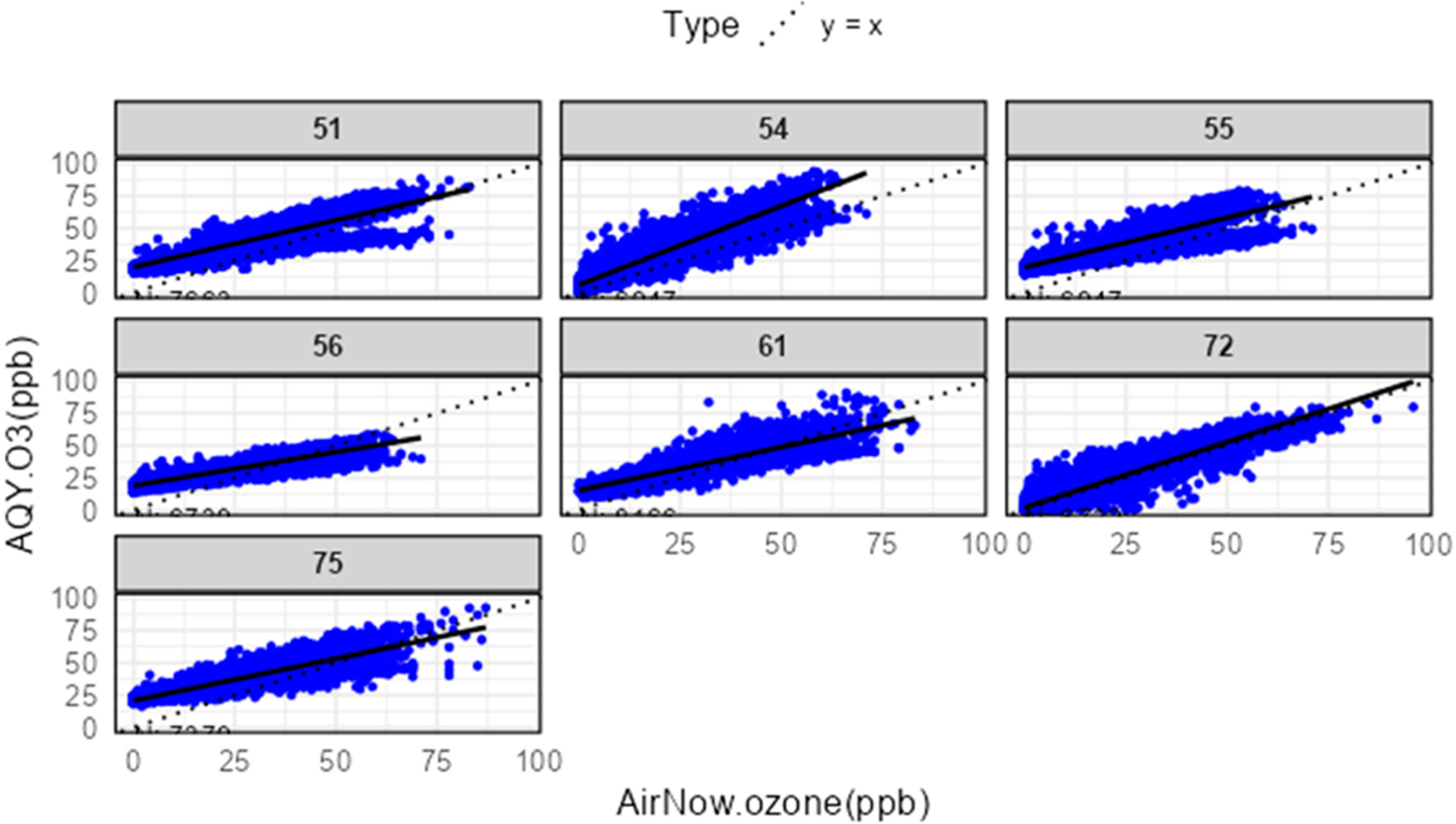
Scatterplots of hourly AQY O_3_ sensor data versus AirNow O_3_ monitor data by sensor ID as generated in ASNAT. The dotted line is the one-to-one line, and the solid black line is the linear regression for each individual sensor. Numbers refer to sensor IDs (Table 1).

Overall, hourly O_3_ sensor measurements were correlated with monitor measurements (R^2^ = 0.70) with a large intercept (Y-Intercept = 14.57 ppb) (Figure 7). Most of the sensors (four out of seven) meet the performance target for O_3_ sensor linearity (R^2^ > 0.8) with other sensors still showing strong correlation (R^2^ ≥ 0.7) (Figure 8). This is stronger linearity than that of past work in Texas where AQY O_3_ sensors had an R^2^ from 0.59 to 0.96 with 3 of 12sensors having an R^2^ < 0.7 [74] but weaker performance than that of previous evaluations in the laboratory (R^2^ = 0.975) and California (R^2^ = 0.96) [29]. Two-prong distributions are seen for sensors 51 (DE, R^2^ = 0.73), 54 (NC, R^2^ = 0.83), and 55 (NC, R^2^ = 0.70), suggesting that there may be a difference in sensor–monitor relationship under distinct conditions in some cases, leading to sensors not meeting the linearity target (Figure 9). For the three sensors collocated in NC (54, 55, 56), intercepts range from 6.13 to 19.7 ppb, and slopes range from 0.52 to 1.22 (Figure 9, Table S3), highlighting that this is likely due to differences in individual sensors (e.g., hardware) and not differences in site conditions across the states. Based on the wide range of slopes and intercepts between sensors, individual sensor corrections are needed to improve performance so that all sensors will meet the slope and intercept performance targets.

Next, we considered whether a more advanced correction would improve sensor performance. We consider linear regression, multivariable additive, and multiplicative interactive corrections using RH and temperature as measured by the sensors and days since deployment. These corrections consider the influence of environmental conditions, which were variable across the sites. Temperature was relatively consistent across sites with higher temperatures in AZ and slightly lower temperatures in CO (Figure S3). RH conditions were more variable with dryer conditions in AZ and CO (Figure S4). Sensor 54 in NC saw higher RH than the other sensors in NC, suggesting some differences in internal RH sensor performance. Considering the days since deployment considers sensor drift over time.

For temperature and RH corrections, correction improves R^2^ by ≤ 0.04, suggesting that the additional variables explain ≤ 4% of the variation between the sensors and the monitor (Table 3). The sensors that do not meet the R^2^ target are still unable to meet the target with these corrections. For the correction using an additive correction for days since deployment, R^2^ improved by 0.01 to 0.11, leading to sensors in DE and NC meeting the linearity target when they did not with other corrections. Using the multiplicative interactive correction for days deployed further improves most sensors, allowing all sensors to meet the target for linearity. Considering the terms in the equations generated (Table S3), the results suggest that the output from most sensors decreases over the project year. This is in line with past work where AQY and other metal oxide sensors were shown to drift over similar time periods [48,75]. To generate accurate data from these sensors, it is likely that users will need individual sensor corrections that account for drift over time or that sensors will need periodic collocation (e.g., quarterly) and correction updates to ensure O_3_ estimates remain accurate.

## 4. Discussion

The limitations of this tool include data size limits, the need for non-EPA users to install the tool locally, and some limitations in functionality. Dataset size limits are dependent on local computing resources. Users may need to limit longer time analysis to smaller spatial ranges and/or longer averaging intervals. Depending on future resources and priorities, we may try to host a public version of the tool to reduce the installation burden and make the tool more accessible to a wider variety of users. Since the code is publicly available, other organizations may also choose to publicly host the tool. There will always be additional analysis, quality assurance, or plots that could be helpful to understand local air quality issues,; however, this tool gives users without data analysis expertise, and those with it, a quick way to perform some helpful analysis.

In addition, more complicated corrections (e.g., machine learning, additional variables) are sometimes required to produce adequate sensor performance [13,34,76], but these more complicated corrections are not currently possible to be made in ASNAT. These more complex corrections can increase the risk of overfitting (i.e., generating a correction model that performs inadequately outside of the calibration period) [77,78], are not easy to interpret [77], and require longer calibration periods [34]. For these reasons, we do not include more complicated corrections currently to hopefully prevent users from generating unhelpful corrections. In the future, these features could potentially be improved through built-in safeguards (e.g., cross-validation), but this is beyond the scope of the current tool.

Some background air quality knowledge is needed to successfully use this tool and make informed decisions based on ASNAT outputs. For example, the nearest neighbor radius will depend on pollutant chemistry, local sources, and geography. Our example focused on pairs within 250 m, but future researchers would need to carefully consider an appropriate distance for comparison. In addition, local knowledge may be required to determine whether outliers are due to sensor malfunctions or real short-term pollutant events.

This project is ongoing, and we hope to add additional features and improvements based on feedback from the initial users. In addition, since the code is open-source and publicly available, we hope users will take it and modify it as needed for their own uses, adding additional functionality and customized displays. So far, more than two hundred air quality professionals have been trained to use this tool including staff from state, local, and tribal agencies; the EPA; other federal agencies; academia; consulting companies; and other organizations. The high engagement in tool training and office hours highlights the strong need for this type of tool.

Our case studies were focused on public PurpleAir data loaded through RSIG and Aeroqual AQY data loaded from local sensor files, but any sensor data that can be put into the ASNAT format can be used in ASNAT in the future, meaning that this tool will be helpful as new sensors enter the market. Currently, many of the large sensor networks in the U.S. are PM_2.5_ sensor networks, but as sensors for additional pollutants become more ubiquitous, this tool can be used to understand performance and local air quality for a variety of different pollutants in addition to PM_2.5_ and O_3_.

The midwestern smoke case study explored the performance of sensors under smoke conditions, but users could consider exploring sensor performance over several different types of localized events (e.g., dust events, fireworks, inversions). Our case study highlights strong performance during regional wildfire smoke impacts. However, seasonal variations in pollutants, pollutant sources, environmental conditions, particle properties, and other factors may lead to seasonal trends in correlation and bias between sensors and monitors [15,79,80] whether considering PM_2.5_ or other pollutants. One of the values of ASNAT is that it allows users to easily generate statistics for different time periods, allowing users to compare seasonal differences in sensor performance. In addition, infrequent or historic events that impact local emissions (e.g., COVID-19 pandemic) can be further analyzed.

The O_3_ sensor performance case study explored the performance of AQY sensors and the influence of temperature and RH and drift on sensor performance. Other sensors may have other influential or interferent variables (e.g., cross-sensitive pollutants), and if those pollutants are measured by the sensor, then those corrections could be considered using ASNAT as well. Some custom code was required for this case study. ASNAT and ASDU are still under development, and we hope that future improvements will limit the need for custom code. However, some custom analysis will likely always be needed for in-depth analysis. This analysis was still more streamlined with a few short chunks of custom code written rather than needing to perform all analysis and determine all relevant plots and statistics to generate without the support of ASDU and ASNAT.

This tool contributes to increasing the utility of air sensor data. For example, state agency staff plan to use the tool to help support a community monitoring project using PurpleAir sensors and to perform regression analysis on multiple PurpleAir sensors collocated with an air monitor. Also, they plan to use the tool to understand how custom-built sensors compare to nearby PurpleAir sensors. Local agency staff plan to use the tool to understand how local sources (e.g., incinerators) impact air quality at schools. By reducing the time it takes to complete analysis, resources are saved that can be put into furthering these projects or other agency priorities. In addition, this data can be used to take informed actions to better protect public health. This may include providing more accurate data to the public or better understanding local sources.

## 5. Conclusions

The development of the Air Sensor Network Analysis Tool (ASNAT) represents a significant advancement in the field of air quality monitoring and analysis. This R-Shiny application addresses key challenges faced by air quality professionals, particularly those related to the integration and analysis of diverse air sensor data. By offering a streamlined, user-friendly platform, ASNAT empowers users to effectively manage, analyze, and visualize air quality data, thereby enhancing their ability to make informed decisions regarding public health and environmental protection.

The case study on Canadian wildfire smoke impacts in the Midwestern United States illustrates ASNAT’s utility in real-world scenarios, demonstrating its capacity to investigate sensor data quality, and it showed that the sensors met performance targets and combined comparable data sources so two discrete sets of air quality measurements could be used together to understand local air quality conditions. Through its comprehensive suite of tools—including data flagging, correction development, and performance evaluation—ASNAT supports the reproducibility and reliability of air quality analyses, ultimately contributing to more accurate public communication and response strategies during air quality events.

The O_3_ case study further underscores the versatility and applicability of ASNAT in diverse air quality monitoring scenarios. By evaluating the performance of Aeroqual AQY O_3_ sensors, ASNAT demonstrated its capacity to handle complex datasets and provide insightful analyses that are crucial for effective air quality management. This study highlighted the tool’s ability to identify variables impacting sensor performance over time and the necessity for individual sensor corrections to maintain data accuracy. By facilitating the development of tailored corrections, ASNAT ensures that air quality professionals can maintain high data integrity across various sensor types and environmental conditions. This adaptability is essential for addressing the dynamic nature of air pollution and supports the ongoing efforts to enhance public health protection. As ASNAT continues to evolve, its role in bridging the gap between raw sensor data and insights will be pivotal in advancing air quality research and monitoring.

Air sensors are becoming ubiquitous, and they represent the next frontier in air quality monitoring globally. We need tools like ASNAT to help the rapidly growing user community. ASNAT is a valuable resource for air quality professionals, enabling more efficient data analysis and supporting efforts to mitigate air pollution exposure. As more air quality professionals adopt ASNAT, its impact is expected to grow, fostering collaborations and innovations that will further advance the field. By facilitating a better understanding and management of air sensor data, ASNAT contributes to the broader goal of protecting public health and the environment. Future enhancements and user-driven modifications will likely expand its capabilities, solidifying its role as a cornerstone tool in air quality monitoring and analysis.

## Supplementary Material

Supplement1

The following supporting information can be downloaded at: https://www.mdpi.com/article/10.3390/atmos16111270/s1, Table S1. Web data that can be loaded in ASNAT through RSIG. Table S2. Available flagging methods [72,73]. Table S3. Models generated in ASNAT for linear, multivariable additive, and multivariable interaction corrections using days since deployed as the 3rd variable. Figure S1. Ozone sensor performance evaluation sites. Figure S2. AirNow monitor concentrations where 0 represents data from all sites. Figure S3. Temperature as measured by AQY sensors. Figure S4. RH as measured by the AQY sensors.

## Figures and Tables

**Table 1.** Summary of O_3_ performance dataset.

State	AirNow Monitor ID	Monitor AQS ID	Monitor Type	Sensor ID	Sensor Dataset Start	Sensor Dataset End	Mean AirNow O_3_ (ppb)	Max AirNow O_3_ (ppb)
AZ	7231	04–013-0019	Teledyne *—400T	72	4 October 2019	23 July 2020	27	96
CO	7722	08–031-0026	Teledyne *—400E	75	7 August 2019	23 July 2020	28	87
DE	7809	10–003-2004	Thermo ^†^—49i	51	14 August 2019	23 July 2020	28	83
NC	8912	37–063-0099	Teledyne *—T265	54, 55, 56	1 August 2019	24 July 2020	27	71
OK	9123	40–109-1037	Teledyne *—T400	61	1 August 2019	31 July 2020	33	83

* Teledyne API (San Diego, CA, USA).

† Thermo Fisher Scientific (Waltham, MA, USA).

**Table 2.** Number of AQS sites and neighbors, locations, and R^2^ for different distances between sensor–monitor pairs. Including R^2^ for all data and for each sensor–monitor data point over full range and excluding higher-concentration points.

Distance (m)	Number of AQS Sites with Nearby Sensors	Neighbors	Locations	R^2^ for All Data	Range of R^2^ By Sensor–Monitor Pair	R^2^ of All Low-Concentration Data (PM_2.5_ ≤ 18 µg/m^3^)	Range of R^2^ for All Low-Concentration Data by Sensor–Monitor Pair (PM_2.5_ ≤ 18 µg/m^3^)
50	5	9	Waterloo, IA Cedar Rapids, IA Des Moines (×2), IA Davenport, IA	0.88	0.80–0.99	0.63	0.48–0.95
250	8	16	Clinton, IA Iowa City, IA Muscatine, IA *	0.87	0.62–0.99	0.62	0.42–0.95
500	11	20	Chicago, IL Cicero, IL Minneapolis, MN *	0.89	0.27–0.99	0.60	0.03–0.95
1000	14	26	Schiller Park, IL Clinton, IA Minneapolis, MN (2× total) ^†^ Madison, WI *	0.94	0.27–0.99	0.67	0.03–0.95
2000	18	48	Kalamazoo, MI Blaine, MN Green Bay, WI Waukesha, WI *	0.92	0.27–1.00	0.62	0.03–1.00
4000	30	107	Naperville, IL Rockford, IL Gary, IN Ogden Dunes (Wickliffe), IN Holland, MI Dearborn, MI Detroit, MI Rochester, MN St Paul, MN Duluth, MN Apple Valley, WI Madison, WI (2× total) ^†^*	0.87	0.27–1.00	0.30	0.03–1.00

* And all locations within previous distance.

† One pair in this city already and one more added at this distance, equaling two sites in this city total.

**Table 3.** R^2^ for various corrections applied (additive “+” and multiplicative interaction “*”) with ASNAT and the improvement in R^2^ that more complex corrections provide from linear correction. Shaded cells meet the performance target (R^2^ ≥ 0.8). Days represents the days since deployment, and T is temperature.

					R^2^					Improvement from Linear				

State	Sensor ID	Linear	+ T	* T	+ RH	* RH	+ Days	* Days	+ T	* T	+ RH	* RH	+ Days	* Days
AZ	72	0.90	0.91	0.92	0.90	0.90	0.91	0.91	0.01	0.02	0.00	0.00	0.01	0.01
CO	75	0.80	0.80	0.80	0.80	0.80	0.86	0.90	0.00	0.00	0.00	0.00	0.06	0.10
DE	51	0.73	0.76	0.77	0.74	0.74	0.81	0.85	0.03	0.04	0.01	0.01	0.08	0.12
NC	54	0.83	0.83	0.84	0.84	0.84	0.90	0.90	0.00	0.01	0.01	0.01	0.07	0.07
NC	55	0.70	0.73	0.74	0.72	0.72	0.81	0.85	0.03	0.04	0.02	0.02	0.11	0.15
NC	56	0.81	0.83	0.83	0.82	0.83	0.85	0.87	0.02	0.02	0.01	0.02	0.04	0.06
OK	61	0.75	0.76	0.76	0.76	0.76	0.78	0.83	0.01	0.01	0.01	0.01	0.03	0.08

All	All avg	0.79	0.80	0.81	0.80	0.80	0.85	0.87	0.01	0.02	0.01	0.01	0.06	0.08

## Data Availability

Code is available on github at https://github.com/USEPA/Air-Sensor-Network-Analysis-Tool-Public-, accessed on 30 September 2025, and operating system-specific zip files are available at the following links: Windows https://ofmpub.epa.gov/rsig/rsigserver?asnat/download/Windows/ASNAT.zip, accessed on 30 September 2025, Mac https://ofmpub.epa.gov/rsig/rsigserver?asnat/download/Darwin.arm64/ASNAT.zip, accessed on 30 September 2025, Mac https://ofmpub.epa.gov/rsig/rsigserver?asnat/download/Darwin.x86_64/ASNAT.zip, accessed on 30 September 2025, and Linux https://ofmpub.epa.gov/rsig/rsigserver?asnat/download/Linux.x86_64/ASNAT.zip, accessed on 30 September 2025.

## Abbreviations

The following abbreviations are used in this manuscript:

API: Application Programming Interface
AQI: Air Quality Index
AQS: Air Quality System
ASDU: Air Sensor Data Unifier
ASNAT: Air Sensor Network Analysis Tool
CFR: Code of Federal Regulations
CO: Carbon Monoxide
csv: Comma-Separated Values
EPA: Environmental Protection Agency
FEM: Federal Equivalent Method
FRM: Federal Reference Method
IA: Iowa
ID: Identification
IL: Illinois
IN: Indiana
MI: Michigan
MN: Minnesota
METAR: Meteorological Aerodrome Report
MTA: Material Transfer Agreement
NO_2_: Nitrogen Dioxide
NAAQS: National Ambient Air Quality Standards
NMBE: Normalized Mean Bias Error
NRMSE: Normalized Root Mean Squared Error
O_3_: Ozone
OH: Ohio
ORD: Office of Research and Development
PM_10_: Particulate Matter 10 µm or Less in Diameter
PM_2.5_: Fine Particulate Matter 2.5 µm or Less in Diameter
QA: Quality Assurance
QC: Quality Control
R^2^: Coefficient of Determination
RH: Relative Humidity
RMSE: Root Mean Squared Error
RSIG: Remote Sensing Information Gateway
SD: Secure Digital
SO_2_: Sulfur Dioxide
UNC: University of North Carolina at Chapel Hill
U.S.: United States
USD: U.S. Dollars
UT: Utah
UTC: Coordinated Universal Time
WI: Wisconsin
