# Supplementary material for: Air Sensor Network Analysis Tool: R-Shiny Application
Source: Atmosphere (Basel). Author manuscript; Available in PMC 2026 Aug 1. (PMC13426646; doi:10.3390/atmos16111270)
Supplement: Supplement1 [file NIHMS2131755-supplement-Supplement1.pdf]

Supplement

Air Sensor Network Analysis Tool: R-Shiny application

Table S1. Web data that can be loaded in ASNAT through RSIG.

Source	Variable	Units	Description
AirNow	pm25	ug/m3	UTC hourly mean surface measured particulate matter (aerosols) 2.5 microns or smaller in diameter in micrograms per cubic meter.
AirNow	pm10	ug/m3	UTC hourly mean surface measured particulate matter (aerosols) 10 microns or smaller in diameter in micrograms per cubic meter.
AirNow	rh	%	UTC hourly mean surface measured relative humidity percentage.
AirNow	temperature	C	UTC hourly mean surface measured air temperature (C).
AirNow	AirNow.pressure	C	UTC hourly mean surface measured atmospheric pressure (hPa).
AirNow	ozone	ppb	UTC hourly mean surface measured ozone (ppb).
AirNow	no2	ppb	UTC hourly mean surface measured nitrogen dioxide (ppb).
AirNow	co	ppm	UTC hourly mean surface measured carbon monoxide (ppm).
AirNow	so2	ppb	UTC hourly mean surface measured sulfur dioxide (ppb).
AQS	pm25	ug/m3	UTC hourly mean surface measured particulate matter (aerosols) 2.5 microns or smaller in diameter in micrograms per cubic meter.
AQS	pm10	ug/m3	UTC hourly mean surface measured particulate matter (aerosols) 10 microns or smaller in diameter in micrograms per cubic meter.
AQS	rh	%	UTC hourly mean surface measured relative humidity in percent.
AQS	temperature	C	UTC hourly mean surface measured air temperature (C).
AQS	pressure	C	UTC hourly mean surface measured atmospheric pressure (hPa).
AQS	ozone	ppb	UTC hourly mean surface measured ozone (ppb).
AQS	no2	ppb	UTC hourly mean surface measured nitrogen dioxide (ppb).
AQS	co	ppm	UTC hourly mean surface measured carbon monoxide (ppm).
AQS	so2	ppb	UTC hourly mean surface measured sulfur dioxide (ppb).
METAR	relativeHumidity	%	Relative humidity (computed using Magnus approximation formula from temperature and dewPoint).
METAR	temperature	C	Air temperature in degrees C.
METAR	seaLevelPress	hPa	Atmospheric pressure equivalent at sea level in hPa.

PurpleAir	pm25_corrected	ug/m3	Outdoor fine particulate matter concentration ($PM_{2.5}$) that is at least 75% complete and corrected for humidity and bias using Equation 1. AB channel outliers are removed as described in the text.
PurpleAir	humidity	%	Sensor-measured relative humidity inside the sensor housing (on average 4% lower than ambient conditions).
PurpleAir	temperature	C	Temperature inside of the sensor housing. On average this is 4C higher than ambient conditions.
PurpleAir	indoor_pm25_corrected	ug/m3	Indoor fine particulate matter concentration ($PM_{2.5}$) that is at least 75% complete and corrected for humidity and bias using Equation 1. AB channel outliers are removed as described in the text.
PurpleAir	indoor_humidity	%	Indoor sensor-measured relative humidity inside the sensor housing (on average 4% lower than ambient conditions).
PurpleAir	indoor_temperature	C	Indoor temperature inside of the sensor housing. On average this is 4C higher than ambient conditions.
PurpleAir	ozone1	ppb	Sensor-measured ozone concentration.
PurpleAir	pm1	ug/m3	Sensor-measured particulate matter not more than 1 micron in diameter on average for channels A and B but excluding downgraded channels and using the CF=1 variant for indoor and the ATM variant for outdoor devices.
PurpleAir	pm1_a	ug/m3	Sensor-measured particulate matter not more than 1 micron in diameter for channel A using the CF=1 variant for indoor and the ATM variant for outdoor devices.
PurpleAir	pm1_b	ug/m3	Sensor-measured particulate matter not more than 1 micron in diameter for channel B using the CF=1 variant for indoor and the ATM variant for outdoor devices.
PurpleAir	pm1_atm	ug/m3	Sensor-measured particulate matter not more than 1 micron in diameter using the ATM variant average for channels A and B but excluding downgraded channels.
PurpleAir	pm1_atm_a	ug/m3	Sensor-measured particulate matter not more than 1 micron in diameter for the channel A ATM variant.
PurpleAir	pm1_atm_b	ug/m3	Sensor-measured particulate matter not more than 1 micron in diameter for the channel B ATM variant.
PurpleAir	pm25	ug/m3	Sensor-measured particulate matter not more than 2.5 microns in diameter on average for channels A and B but excluding downgraded channels and using the CF=1 variant for indoor and the ATM variant for outdoor devices.
PurpleAir	pm25_a	ug/m3	Sensor-measured particulate matter not more than 2.5 microns in diameter for channel A using the CF=1 variant for indoor and the ATM variant for outdoor devices.
PurpleAir	pm25_b	ug/m3	Sensor-measured particulate matter not more than 2.5 microns in diameter for channel B using the CF=1

			variant for indoor and the ATM variant for outdoor devices.
PurpleAir	pm25_atm	ug/m3	Sensor-measured particulate matter not more than 2.5 microns in diameter using the ATM variant average for channels A and B but excluding downgraded channels.
PurpleAir	pm25_atm_a	ug/m3	Sensor-measured particulate matter not more than 2.5 microns in diameter for the channel A ATM variant.
PurpleAir	pm25_atm_b	ug/m3	Sensor-measured particulate matter not more than 2.5 microns in diameter for the channel B ATM variant.
PurpleAir	pm25_cf_1	ug/m3	Sensor-measured particulate matter not more than 2.5 microns in diameter using the CF=1 variant average for channels A and B but excluding downgraded channels.
PurpleAir	pm25_cf_1_a	ug/m3	Sensor-measured particulate matter not more than 2.5 microns in diameter for the channel A CF=1 variant.
PurpleAir	pm25_cf_1_b	ug/m3	Sensor-measured particulate matter not more than 2.5 microns in diameter for the channel B CF=1 variant.
PurpleAir	pm25_10minute	ug/m3	Sensor-measured particulate matter not more than 2.5 microns in diameter for the 10-minute pseudo-average using the CF=1 data variant for indoor and the ATM variant for outdoor average for channels A and B but excluding downgraded channels.
PurpleAir	pm25_10minute_a	ug/m3	Sensor-measured particulate matter not more than 2.5 microns in diameter for the 10-minute pseudo-average using the CF=1 data variant for indoor and the ATM variant for outdoor channel A running average.
PurpleAir	pm25_10minute_b	ug/m3	Sensor-measured particulate matter not more than 2.5 microns in diameter for the 10-minute pseudo-average using the CF=1 data variant for indoor and the ATM variant for outdoor channel B running average.
PurpleAir	pm25_60minute	ug/m3	Sensor-measured particulate matter not more than 2.5 microns in diameter for the 60-minute pseudo-average using the CF=1 data variant for indoor and the ATM variant for the outdoor average for channels A and B but excluding downgraded channels.
PurpleAir	pm25_60minute_a	ug/m3	Sensor-measured particulate matter not more than 2.5 microns in diameter for the 60-minute pseudo-average using the CF=1 data variant for indoor and the ATM variant for outdoor channel A running average.
PurpleAir	pm25_60minute_b	ug/m3	Sensor-measured particulate matter not more than 2.5 microns in diameter for the 60-minute pseudo-average using CF=1 data variant for indoor and ATM variant for outdoor channel B running average.
PurpleAir	pm10	ug/m3	Sensor-measured particulate matter not more than 10 microns in diameter average for channels A and B but excluding downgraded channels and using the CF=1 variant for indoor and the ATM variant for outdoor devices.

PurpleAir	pm10_a	ug/m3	Sensor-measured particulate matter not more than 10 microns in diameter channel for the A CF=1 variant for indoor and the ATM variant for outdoor devices.
PurpleAir	pm10_b	ug/m3	Sensor-measured particulate matter not more than 10 microns in diameter for the channel B CF=1 variant for indoor and the ATM variant for outdoor devices.
PurpleAir	pm10_atm	ug/m3	Sensor-measured particulate matter not more than 10 microns in diameter for the ATM variant average for channels A and B but excluding downgraded channels.
PurpleAir	pm10_atm_a	ug/m3	Sensor-measured particulate matter not more than 10 microns in diameter for the channel A ATM variant.
PurpleAir	pm10_atm_b	ug/m3	Sensor-measured particulate matter not more than 10 microns in diameter for the channel B ATM variant.
PurpleAir	pm10_cf_1	ug/m3	Sensor-measured particulate matter not more than 10 microns in diameter for the CF=1 variant average for channels A and B but excluding downgraded channels.
PurpleAir	pm10_cf_1_a	ug/m3	Sensor-measured particulate matter not more than 10 microns in diameter for the channel A CF=1 variant.
PurpleAir	pm10_cf_1_b	ug/m3	Sensor-measured particulate matter not more than 10 microns in diameter for the channel B CF=1 variant.
PurpleAir	0_3_um_count	particles/100ml	Count concentration of all particles greater than 0.3 microns in diameter for the average particle count for channels A and B but excluding downgraded channels.
PurpleAir	0_3_um_count_a	particles/100ml	Count concentration of all particles greater than 0.3 microns in diameter for channel A.
PurpleAir	0_3_um_count_b	particles/100ml	Count concentration of all particles greater than 0.3 microns in diameter for channel B.
PurpleAir	0_5_um_count	particles/100ml	Count concentration of all particles greater than 0.5 microns in diameter for the average particle count for channels A and B but excluding downgraded channels.
PurpleAir	0_5_um_count_a	particles/100ml	Count concentration of all particles greater than 0.5 microns in diameter for channel A.
PurpleAir	0_5_um_count_b	particles/100ml	Count concentration of all particles greater than 0.5 microns in diameter for channel B.
PurpleAir	1_um_count	particles/100ml	Count concentration of all particles greater than 1 micron in diameter for the average particle count for channels A and B but excluding downgraded channels.
PurpleAir	1_um_count_a	particles/100ml	Count concentration of all particles greater than 1 micron in diameter for channel A.
PurpleAir	1_um_count_b	particles/100ml	Count concentration of all particles greater than 1 micron in diameter for channel B.
PurpleAir	2_5_um_count	particles/100ml	Count concentration of all particles greater than 2.5 microns in diameter for the average particle count for channels A and B but excluding downgraded channels.
PurpleAir	2_5_um_count_a	particles/100ml	Count concentration of all particles greater than 2.5 microns in diameter for channel A.

PurpleAir	2_5_um_count_b	particles/100ml	Count concentration of all particles greater than 2.5 microns in diameter for channel B.
PurpleAir	5_um_count	particles/100ml	Count concentration of all particles greater than 5 microns in diameter for the average particle count for channels A and B but excluding downgraded channels.
PurpleAir	5_um_count_a	particles/100ml	Count concentration of all particles greater than 5 microns in diameter channel A.
PurpleAir	5_um_count_b	particles/100ml	Count concentration of all particles greater than 5 microns in diameter for channel B.
PurpleAir	10_um_count	particles/100ml	Count concentration of all particles greater than 10 microns in diameter for the average particle count for channels A and B but excluding downgraded channels.
PurpleAir	10_um_count_a	particles/100ml	Count concentration of all particles greater than 10 microns in diameter for channel A.
PurpleAir	10_um_count_b	particles/100ml	Count concentration of all particles greater than 10 microns in diameter for channel B.
PurpleAir	pressure	hPa	Sensor-measured atmospheric pressure.
PurpleAir	voc	IAQ	Sensor-measured volatile organic compounds in Bosch static IAQ units as per the BME680 spec sheet (EXPERIMENTAL).

Table S2. Available flagging methods.

<table>
<tr><th>Flag type</th><th>Description</th><th>Explanation and Examples</th></tr>
<tr><td>Manual</td><td>Users can select individual rows in the interactive table and apply flag “99”.</td><td>Users can click on a row then click the buttons labeled ‘Include 99 in flagged column’ or ‘Exclude 99 from flagged column’ to manually flag/unflag data.</td></tr>
<tr><td>User-specified Boolean expressions</td><td>Users can type flag conditions which are Boolean expressions (i.e., logical statements evaluated as either true or false) into a text box entry in the tool and then press the “Apply Flagging Conditions Above” or “Clear Flagging” button.</td><td>Can be applied to all the available columns of a dataset and are evaluated on each data line. If true, they include an integer code for each true flag condition (i.e., 1 for the first condition, 2 for the second condition, and so on). The column names are operands.

Available relational operators are as follows: <=, >, >=, =, !=, or, and (,). Available R operators are as follows: ||, &&, -, +, *, /, ^, etc.

Note * and 'and' have higher precedence than + and 'or', and ^ is right-associative (e.g., 2 + 3 * 4 = 14 and 2^3^2 = 512). Use parentheses only when necessary.

“#” Indicate comments.

Examples:
#flag condition 1
(id = 44275 or id = 99449) and count < 25
#flag condition 2
pm25_corrected_hourly < 0
#flag condition 3
2022-06-01T00 <= timestamp and timestamp <= 2022-06-01T05</td></tr>
<tr><td>User-selected—Dataset Y neighbor flagging</td><td>Apply flag 80 if difference > (user-fillable box).

Apply flag 81 if percent difference > (user-fillable box).

Apply flag 82 if R-squared < (user-fillable box).</td><td>Data from X/Y neighbor pairs are examined, and either individual data points (flags 80 and 81) or all data from the X/Y pair (flag 82) are flagged based on the relationship between the datasets.</td></tr>
<tr><td>User-selected—</td><td>Apply flag 83 if a constant value persists for at least x (user-defined)</td><td>Flag 83—Constant value: For each station, this flags the data if at least x (user-defined) consecutive identical values occur.</td></tr>
</table>

Additional flags	consecutive measurements.<br><br>Apply flag 84 if missing data for at least x (user-defined) consecutive measurements.<br><br>Apply flag 85 if data point is a statistical outlier.<br><br>Apply outlier detection within a specified time window.<br><br>Apply flag 86 if data point is an outlier by the Hampel filter.	Example: Values [5,5,5,6] with x=3 → first three rows flagged 83.<br><br>Flag 84—Long missing data: For each station, this flags the data if at least x (user-defined) consecutive missing values occur.<br><br>Example: Values [NA,NA5,6] with x=2 → first two rows flagged 84.<br><br>Flag—85 Statistical outlier: Per station, this flags rows where ∣value − mean∣ > k * standard deviation (k = outlier_threshold specified by the user). If a time window is enabled, it only flags within [start, end].<br><br>Example: Mean=20, SD=4, k=2; value=29 → ∣9∣>8 → flag 85.<br><br>Can provide a specific time window to apply the outlier detection method.<br><br>Flag 86—The Hampel filter is an outlier detection method based on the median and median absolute deviation (MAD). It identifies outliers by replacing each observation with the median of its neighborhood and flagging points that deviate beyond a multiple of the MAD [70,71].<br><br>Hampel filter outlier: A rolling Hampel is applied with window n and threshold t; rows with score > t are flagged 86.
User-selected—Invalid values flags	Apply flag 60 for invalid negative values in pollutant measurements (O_3, NO_2, CO, etc.).	Removes negative values. In some cases, negative values should be left in if they are due to noise so that longer-term averages are not biased high or so baseline shifts are not hidden.
User-selected—Cross-field validation flags	Apply flag 65 for temporal inconsistencies (e.g., end date before start date).	This flag is applied to records where timestamps are not in chronological order. If a device's first timestamp is later than its last, all rows for that device are flagged with 65. Otherwise, only the adjacent non-chronological data points are flagged.<br><br>Examples:<br><br>End date before start date: first=2022-06-05T00:00:00-0000; last=2022-06-01T00:00:00-0000 → all rows for that device are flagged 65.<br><br>Non-chronological: … 2022-06-01T01:00:00-0000, 2022-06-01T00:30:00-0000 … → these two rows are flagged 65.

Suspicious changes flags	Apply flag 70 for sudden spikes in pollutant levels (user-fillable box for window and threshold).<br><br>Apply flag 71 for sudden drops in pollutant levels (user-fillable box for window and threshold).<br><br>Apply flag 72 for inconsistent daily ozone patterns.<br><br>Apply flag 73 for PM inconsistent daily patterns.	Flag 70—Sudden spikes: Compares the current value to the mean of the previous W values of the selected variable (per station). If (current − prev_avg)/prev_avg > threshold, flag 70 is added.<br><br>Example: Prev= [10,12,8] → avg=10; current=16; threshold=0.5 → (16−10)/10=0.6>0.5 → flag 70.<br><br>Flag 71—Sudden drop: If (prev_avg − current)/prev_avg > threshold, 71 is added.<br><br>Example: Prev_avg≈50; current=25; threshold=0.4 → (50−25)/50=0.5>0.4 → flag 71.<br><br>Flag 72—Inconsistent daily ozone pattern: The daytime (06:00–18:00) median and standard deviation are computed; nighttime (00:00–05:00) ozone values that exceed *daytime_median + 1.5 × daytime_sd* are flagged.<br><br>Example: Day median=40, SD=10; night value=58 → 40 + 1.5 × 10 = *55;* → 58>55 → flag 72.<br><br>Flag 73—PM inconsistent daily pattern: Afternoon (15–17) PM should be lower; afternoon values above morning_median + 1.5*morning_sd or above night_median + 1.5*night_sd (morning 07–10; night 21–23) are flagged.<br><br>Example: Morning median=25, SD=5; afternoon=35 → 25 + 1.5 x 5 = 32.5 → 35>32.5 → flag 73.
Redundancy check	Apply flag 90 for duplicate timestamps at the same location.	This flag is applied to records that share the same timestamp and location. Duplicates are defined by timestamp (UTC) + longitude(deg) + latitude(deg) (and elevation(m) if available). All rows in each duplicate set are flagged with 90.<br><br>Examples: Duplicate pair: two rows at 2022-06-01T00:00:00-0000, (lat, lon) = (35.123, -80.456) → both flagged 90.
Invalid formatting and units	Apply flag 95 for incorrect date formats.<br><br>Check for inconsistent units across variables and flag problematic variables.	Flag 95—Incorrect date formats: Flags rows where the timestamp (UTC) does not match YYYY-MM-DDTHH:MM:SS-0000.<br><br>Example: 2022/06/01 00:00 → flag 95.<br><br>Unit consistency check (UI): Lists variables with unexpected units (e.g., longitude not in deg, RH not in %). Shown in a table; no row flags assigned.

		Example: longitude(rad) or ozone(ug/m3) appear in “Inconsistent Variables.”

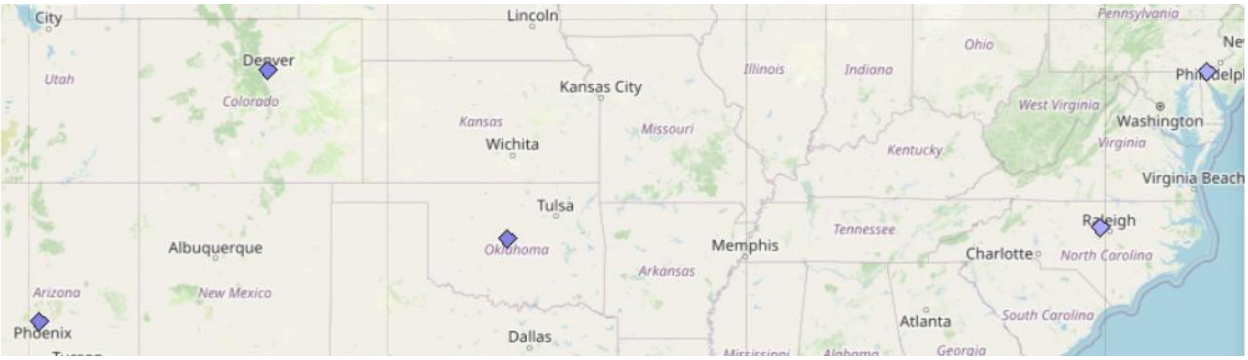

Figure S1. Ozone sensor performance evaluation sites.

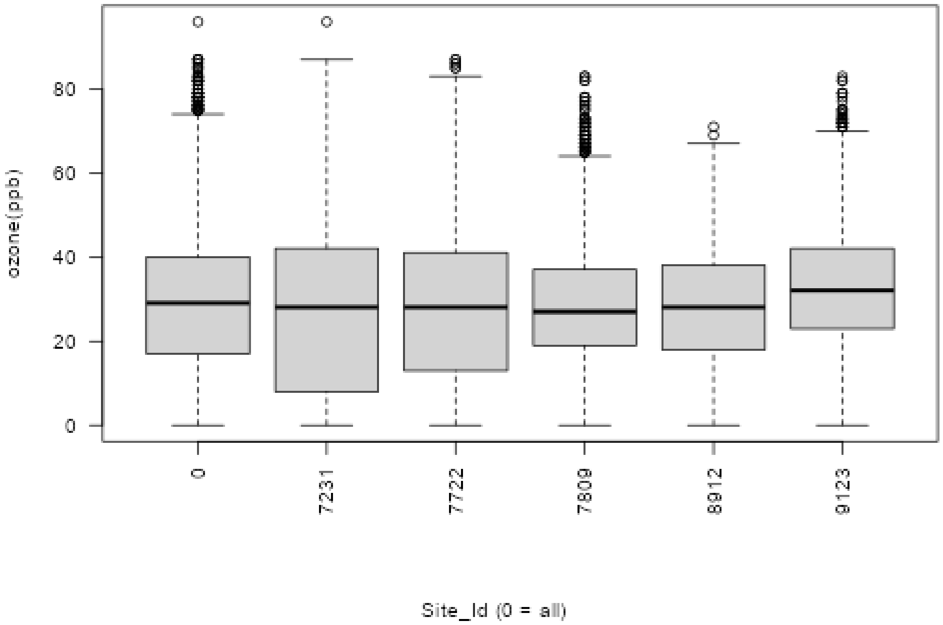

Figure S2. AirNow monitor concentrations where 0 represents data from all sites.

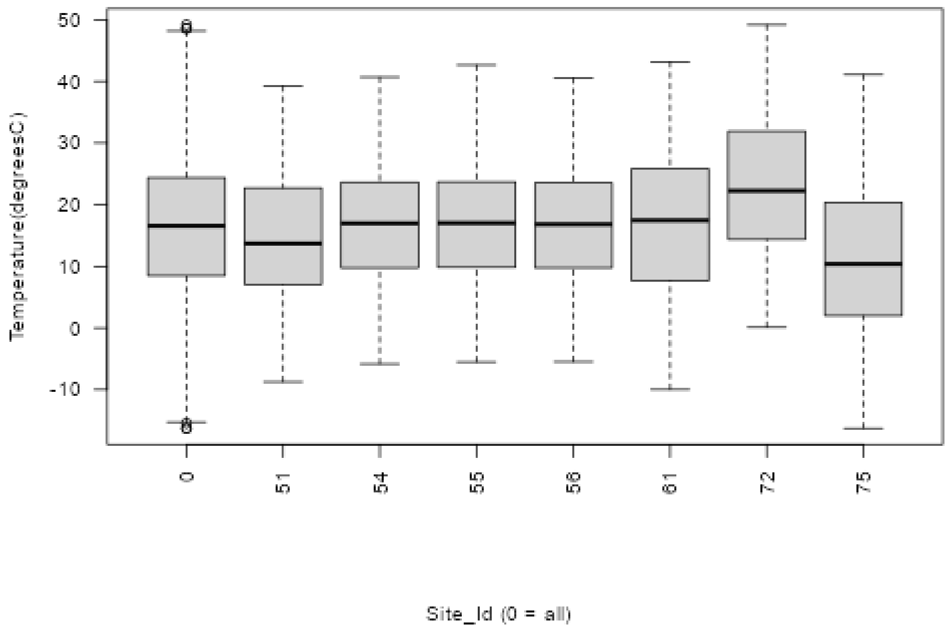

Figure S3. Temperature as measured by AQY sensors.

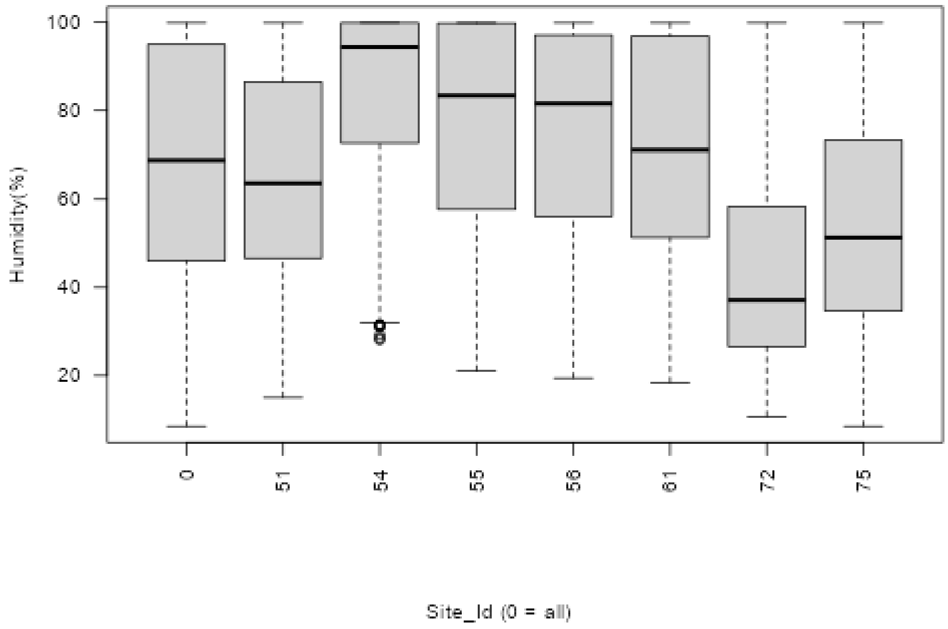

Figure S4. RH as measured by the AQY sensors.

Table S3. Models generated in ASNAT for linear, multivariable additive, and multivariable interaction corrections using days since deployed as the 3rd variable.

Sensor ID	Model Type	Intercept	Coefficient *AQY O3	Coefficient*Days	Coefficient*AQY O3* Days
72	Linear	1.9127	1.0176	-	-
	+days since start	-3.1087	0.9686	0.0286	-
	*days since start	-8.229	1.1747	0.0553	-0.001
75	Linear	20.7164	0.6519	-	-
	+days since start	16.1596	0.6103	0.0323	-
	*days since start	24.0411	0.3676	-0.0186	0.0015
51	Linear	19.9171	0.7259	-	-
	+days since start	15.0298	0.6524	0.0368	-
	*days since start	23.7874	0.3579	-0.014	0.0016
54	Linear	6.1317	1.2238	-	-
	+days since start	-1.6846	1.1741	0.0497	-
	*days since start	3.0248	1.0085	0.0211	0.001
55	Linear	19.7149	0.7674	-	-
	+days since start	12.8146	0.7237	0.0438	-
	*days since start	21.583	0.4155	-0.0094	0.0018
56	Linear	18.8907	0.5229	-	-
	+days since start	16.1385	0.506	0.0169	-
	*days since start	20.509	0.3527	-0.0089	0.0009
61	Linear	15.2396	0.6724	-	-
	+days since start	12.6142	0.6578	0.0174	-
	*days since start	22.7861	0.3723	-0.0431	0.0017
